# Precision-engineered metal–organic frameworks: fine-tuning reverse topological structure prediction and design†

**DOI:** 10.1039/d4sc05616g

**Published:** 2024-09-25

**Authors:** Xiaoyu Wu, Jianwen Jiang

**Affiliations:** a Department of Chemical and Biomolecular Engineering, National University of Singapore 117576 Singapore chejj@nus.edu.sg

## Abstract

Digital discoveries of metal–organic frameworks (MOFs) have been significantly advanced by the reverse topological approach (RTA). The node-and-linker assembly strategy allows predictable reticulations predefined by *in silico* coordination templates; however, reticular equivalents lead to substantial combinatorial explosion due to the infinite design space of building units (BUs). Here, we develop a fine-tuned RTA for the structure prediction of MOFs by integrating precise topological constraints and leveraging reticular chemistry, thus transcending traditional exhaustive trial-and-error assembly. From an extensive array of chemically realistic BUs, we subsequently design a database of 94 823 precision-engineered MOFs (PE-MOFs) and further optimize their structures. The PE-MOFs are assessed for post-combustion CO_2_ capture in the presence of H_2_O and top-performing candidates are identified by integrating three stability criteria (activation, water and thermal stabilities). This study highlights the potential of synergizing PE with the RTA to enhance efficiency and precision for computational design of MOFs and beyond.

## Abstract 2

A fine-tuned reverse topological approach is developed for structure prediction and 94 823 precision-engineered metal–organic frameworks are designed.

## Graphical abstract

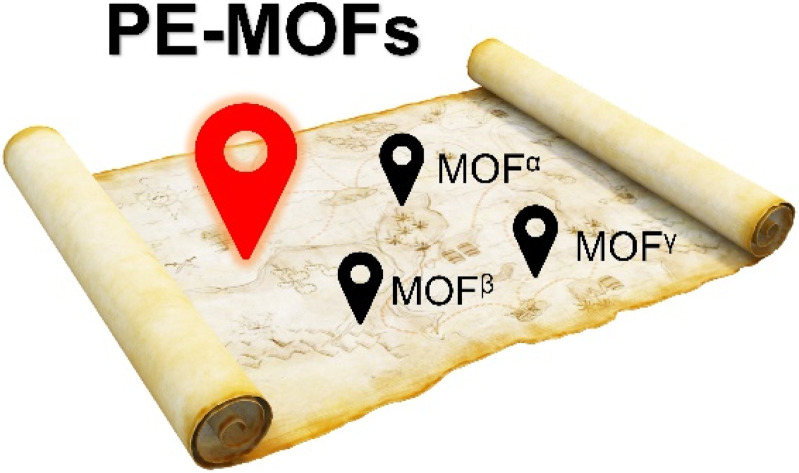

## 1. Introduction

Porous crystals such as metal–organic frameworks (MOFs) represent a significant advancement in the field of functional materials, distinguished by their modular architectures and diverse chemical functionalities.^1^ With these salient features, MOFs are considered versatile materials for many potential applications including gas storage, separation, catalysis, *etc.*^2^ The evolution of MOF landscape has been remarkably enriched by the fusion of experimental synthesis and theoretical analysis, particularly through digital reticular chemistry.^3^

Experimentally synthesized MOFs, through systematically orchestrated assembly of metal nodes and organic linkers, have provided tangible crystals for characterization and validation. Meanwhile, theoretical methods, facilitated by automated assembly,^4^*ab initio* crystal structure prediction,^5,6^ building unit (BU) replacement,^7^ and predominantly, the reverse topological approach (RTA),^8^ have exponentially expanded the conceivable landscape of MOFs and generated extensive hypothetical MOFs (hMOFs) without physical synthesis.^9–11^ The RTA operates by starting with a desired topology template and then mapping it back to specific BUs based on connectivity patterns and topological optimization. Consequently, high-throughput computational screenings have been conducted on experimental MOFs or customized hMOFs and shortlisted potential candidates for many applications, particularly CO_2_ capture.^12–15^ Yet, as the field progresses, a major challenge persists in combinatorial explosion due to a wide variety of BUs and their enormous arrangements. This explosion in structural possibilities, while broadening the horizon of MOFs, imposes a substantial challenge in computation.

**Scheme 1:**
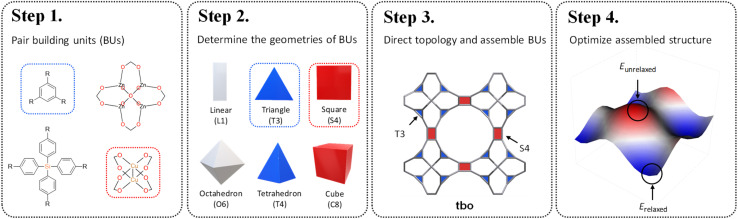
Topological structure prediction with precision engineering. Step 1: pair building units; Step 2: determine the geometries of building units; Step 3: direct topology and assemble building units; Step 4: optimize assembled structure.

Reticular chemistry, a discipline that artfully weaves the threads of molecular architectures, offers an optimized palette of targeted nets controlled by the geometric and topological preference of BUs.^16^ By integrating topological constraints,^17^ one can specifically generate certain crystal configurations based on the geometric compatibility of BU combinations. Inspired by these benefits, in this work, we develop a fine-tuned RTA by introducing an additional layer of precision prerequisite. This refinement involves determining the geometries of BUs and selecting appropriate coordination templates guided by the reticular nature of MOFs. These criteria are governed by the local symmetry of BUs reticulated within a designated net. As illustrated in Scheme 1, our method starts with the one-on-one pairing of organic (red) and inorganic (blue) BUs, where their geometric signatures are subsequently matched and assigned. This approach strategically focuses on identifying a ‘reticular ideal’ topology, thereby alleviating the need for an exhaustive search across the entire topology space. The added dimension of precision engineering (PE) effectively mitigates the combinatorial explosion by filtering out infeasible topologies at an early stage, thereby catalyzing a substantially more focused and accurate search for synthesizable MOFs given a vast array of BUs.

Prompted by the combinatorial guarantee demonstrated above, we present how large-scale RTA-based structure prediction of MOFs can be fine-tuned by PE for vast recursive inorganic–organic combinations of BUs, sourced from the well-established HEALED library.^18^ To ensure a consistent basis for geometry determination, the connection points and the planarity of each candidate BU were standardized, followed by a similarity comparison against established geometric prototypes. Subsequently, we consulted a specialized table of topological constraints to specifically select topologies suited to the selected BUs before *in silico* synthesis and curation. Finally, a total of 94 823 PE-MOFs were distilled and carefully optimized by integrating the universal force field (UFF) and machine-learned atomic charges. The applicability of the PE-MOFs was demonstrated as adsorbents for post-combustion CO_2_ capture. Top-performing candidates were identified and quantitative structure–performance relationships were established. These endeavors not only enrich the existing MOF bank, but also set the stage for future advancement in digital discovery of MOFs. The systematic approach outlined here showcases the potential for computational construction and high-throughput discovery of superior MOFs, ensuring that the next generation of porous crystals will be precisely tailored for specific industrial applications such as CO_2_ capture and beyond.

## 2. Methodology

### 2.1. Geometric signatures

**Fig. 1:**
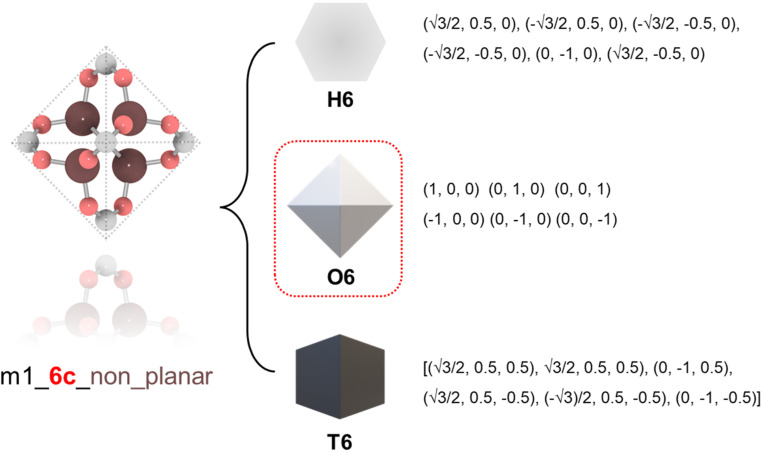
Classification of a BU namely m1 into different geometric prototypes with mathematical representations: hexagonal (H6), octahedral (O6), and trigonal prism (T6). The BU is first identified to be non-planar 6-connected thus excluding H6, and then it is computed with the smallest RMSD against O6 and T6; finally, O6 is determined as the geometric signature.

To ensure precise classification and compatibility check, we defined a comprehensive set of 13 geometric prototypes including 2-connected linear (L2), 3-connected triangle (T3), 4-connected square (S4), 4-connected tetrahedral (T4), 6-connected hexagonal (H6), 6-connected octahedral (O6), 6-connected trigonal prism (T6), 8-connected cubic (C8), 12-connected cuboctahedra (C12), 12-connected icosahedral (I12), 12-connected hexagonal prism (H12), 12-connected truncated tetrahedral (T12), and 24-connected truncated octahedron (T24). Classification of BUs began by determining the number of connection points, followed by evaluating the planarity where applicable, specifically for L2, T3, T4, S4, H6, C8, and T24 geometries. For instance, a BU with 3 connection points was directly classified as T3; a BU with 4 connection points was designated as tetrahedral if it failed the conservative planarity check. Should a BU not conform to these preliminary classifications, further steps would compare it with the detailed geometric prototypes by examining the root-mean squared deviations (RMSDs).^19^ An example is illustrated in Fig. 1 where a BU namely m1 from the HEALED library is assigned with an O6 geometric signature. However, it is worthwhile to note that there are challenges in classifying non-perfectly shaped BUs and highly connected BUs, particularly those with 12 or more connections. These BUs may not always align precisely with desired geometric signatures, even with thorough visual inspections. For instance, Fig. S1† demonstrates how a highly connected vertice BU, namely m345 (sourced from a MOF with **fcu** topology^20^), combined with an edge BU o13, leads to a structure that, while identical in coordination, diverges in linker shape due to the steric effects of the naphthalene linker. In cases where a BU exhibits an imperfect geometric signature, it can still be reticulated into a topology analogous to a perfect BU. As depicted in Fig. S2,† despite imperfect geometry, o165 (o255) can still assemble into **edc** (**ftw**) topology with m345, similar to o189 (o47) with an ideal BU. This adaptability enables the navigation of a broader topological diversity, more aligned with real-world scenarios where perfectly shaped BUs are less common, compared to methods that rely strictly on idealized BUs (Fig. S3†). The full list of the geometric prototypes mathematically constructed in this work is provided in the ESI.†

### 2.2. Topological constraints

**Fig. 2:**
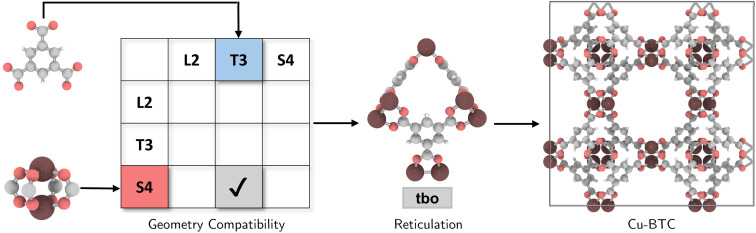
Selection of a compatible topology for the assembly of Cu paddlewheel (Cu_2_O_8_) and 1,3,5-benzene tricarboxylate (BTC), followed by reticulation into a ‘reticular ideal’ **tbo** topology, constructing Cu-BTC.

The coordination complexity of MOF structures arises from the wide variety of possible geometric signatures.^17^ By carefully selecting BUs with appropriate shapes, topological constraints can be targeted. We introduced a reticular reference table for binary combinations of geometric signatures (ESI†), providing information on the most ‘reticular ideal’ topology to form. For instance, Cu-paddlewheel (Cu_2_O_8_) and 1,3,5-benzene tricarboxylate (BTC) can be assigned geometric signatures as T3 and S4, respectively. While conventional connectivity-based methods^10,21,22^ would allow these BUs to assemble into several topologies such as **ctn**, **bor**, **pto**, and **tbo** (Fig. S4†), not all these combinations result in practical or favorable structures due to potential geometric distortions in structure prediction (Fig. S5†). Considering the shape compatibility of BUs, we further refined the selection of topologies, only targeting geometrically coherent structures, and constructed precise and ideal structures such as Cu-BTC^23^ or HKUST-1 (Fig. 2). It is important to note that certain topologies are absent from the publicly available topology database under the RCSR project^24^ and, as such, were not included in our table.

### 2.3. Topological assembly

With topological constraints, the molecular LEGO-like PORMAKE algorithm^10^ was implemented for *in silico* assembly utilizing BUs from the HEALED library.^18^ A total of 200 875 MOFs were initially generated with a RMSD cut-off at 0.3 Å. These structures were distilled to 94 823 PE-MOFs by employing a timeout-based strategy as previously used for computationally curating hMOFs^11^ and covalent–organic frameworks (COFs).^25^ The 94 823 PE-MOFs were structurally optimized by using the SMART algorithm of Forcite module in Materials Studio. The UFF^26^ and machine-learned PACMOF atomic charges^27^ were adopted to describe bonded and non-bonded interactions. In the calculations of atomic charges, a five-minute timeout was implemented to exclude structures being difficult to featurize for PACMOF. The bond information of both inter- and intra-BUs predefined using PORMAKE was preserved during optimizations to avoid unreasonable distance-based bond calculations, which might be caused by improperly configured BUs while fitting into selected topologies. During optimization, a twenty-minute wall-time limit was set to filter out structures that failed to converge. For each structure, bond information was retained and charge information was recalculated. The charge-recalculation and geometric optimization loop were repeated three times to ensure the accuracy of the final set of PE-MOF structures, with a wall-time limit consistently applied throughout these stages.

### 2.4. Diversity analysis

Geometric and chemical features were used for diversity analysis of the designed PE-MOFs. The geometric features including pore limiting diameter (PLD), largest cavity diameter (LCD), channel dimensionality (Dimen), pore volume (PV), void fraction (VF), gravimetric surface area (GSA) and global cavity diameter (GCD) were computed *via* Zeo++ ^28^ utilizing a probe radius of 1.86 Å. The diverse chemical features were encoded using revised autocorrelations (RACs),^29^ which were initially developed to fingerprint open-shell transition metal complexes^30,31^ and have been adapted to encode MOFs.^32–35^ The RACs were computed *via* MolSimplify,^36^ providing the product or difference of atomic heuristics based on non-weighted crystal graphs. A wide variety of properties such as Pauling electronegativity (*χ*), nuclear charge (*Z*) connectivity (*T*), atom identity (*I*), covalent radii (*S*) and polarizability (*α*) were included in the RACs. Disparity (*D*), variety (*V*) and balance (*B*) were computed as diversity metrics^32^ based on unsupervised machine-learned 2D embeddings of the RACs mapped by *t*-distributed Stochastic Neighbor Embedding (*t*-SNE).^37^

### 2.5. Molecular simulation for CO_2_ capture

The PE-MOFs were assessed as adsorbents for CO_2_ capture from a flue gas by pressure-swing adsorption. The flue gas was mimicked using a CO_2_/N_2_ mixture with a composition of 0.15/0.85. Considering the kinetic diameters of CO_2_ (3.30 Å) and N_2_ (3.64 Å), a subset of 17 173 PE-MOFs with PLDs ranging from 3 to 7 Å was chosen. Grand-canonical Monte Carlo (GCMC) simulations were conducted to compute the adsorption of the flue gas at 298 K and 1 bar and the desorption of pure CO_2_ at 298 K and 0.01 bar. The PE-MOFs with performance superior to zeolite-13X^38^ were shortlisted and their water, thermal and activation stabilities were evaluated using MOFSimplify.^39,40^ For the stable ones, their CO_2_ capture performance was further evaluated in the presence of H_2_O. GCMC simulations were extended for the adsorption of a ternary mixture of CO_2_, N_2_ and H_2_O at 298 K and 1 bar, with 60% relative humidity. CO_2_ and N_2_ were represented using the transferable potentials for phase equilibria (TraPPE) force field,^41^ and H_2_O was mimicked by the four point TIP4P model.^42^ The Lennard-Jones interactions of framework atoms were modeled using the UFF^26^ and DREIDING force field.^43^ Table S1† lists the force field parameters. For cross LJ interactions, the potential parameters were estimated using the Lorentz–Berthelot mixing rules. The atomic charges of PE-MOFs were assigned using PACMOF,^27^ which efficiently estimates charges with comparable accuracy to the density derived electrostatic and chemical (DDEC) method.^44^ As exemplified in Fig. S6,† a determination coefficient (*R*^2^) of 0.933 was achieved by comparing PACMOF charges and DDEC charges in four representative PE-MOFs. Following the DDEC6 protocol,^45^ the DDEC charges here were computed using the CHARGEMOL package (https://github.com/berquist/chargemol) based on the calculations from the Vienna *Ab initio* Simulation Package (VASP)^46,47^ at the PBE-D3(BJ) level.^48–50^ Fig. S7† also shows CO_2_, N_2_ and H_2_O uptakes (at 60% relative humidity) simulated using PACMOF charges and DDEC charges, respectively, in the four PE-MOFs. The uptakes based on both charges are close, suggesting an insignificant effect of the charge assignment method. During GCMC simulations, the frameworks were treated as rigid with periodic boundary conditions applied to all three dimensions. The LJ interactions were calculated with a cutoff of 12 Å, while the electrostatic interactions were estimated using Ewald summation. The GCMC simulations were carried out using the RASPA package.^51^ Each simulation included 10 000-cycle equilibration and 10 000-cycle production. Five different trial moves including insertion, deletion, rotation, translation and identity change were attempted randomly with equal probability.

## 3. Results and discussion

### 3.1. Analysis of PE-MOFs

**Fig. 3:**
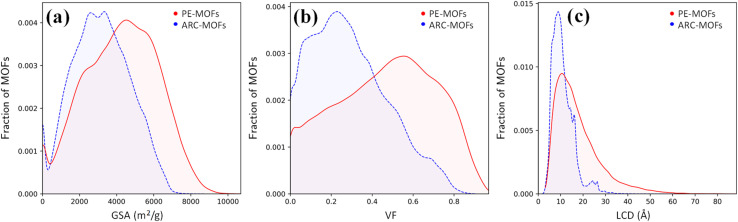
Distributions of (a) GSA, (b) VF and (c) LCD in 94 823 PE-MOFs (red) and 279 010 ARC-MOFs (blue).^14^

We start with the geometric analysis of the PE-MOF database. Fig. 3 shows the distributions of three key geometric features (GSA, VF and LCD) in 94 823 PE-MOFs and also in 279 010 ARC-MOFs for comparison. The ARC-MOF database is a benchmark collection of both experimentally synthesized and computationally predicted MOF deposits.^14^ For each geometric feature, a similar ‘volcano’ pattern is observed in both databases; however, PE-MOFs exhibit a wider distribution. Specifically, GSA ranges from zero to 10 000 m^2^ g^−1^ in PE-MOFs and from zero to 8000 m^2^ g^−1^ in ARC-MOFs. The VF is between 0 and 1 in PE-MOFs and between 0 and 0.9 in ARC-MOFs. The LCD is from 2 Å to 80 Å in PE-MOFs and from 2 Å to 35 Å in ARC-MOFs. These results suggest that our fine-tuned RTA is capable of generating MOFs with a more variety of geometries. Fig. S8 and S9† provide a comparative analysis of topology distribution and its correlation with pore features between PE-MOFs and ARC-MOFs. Despite PE-MOFs being designed with a limited set of BUs, the enriched diversity in topologies significantly influences their porosity profiles, enhancing the variety of geometric features. Fig. S10† illustrates the *t*-SNE map with embeddings of geometric features for PE-MOFs and ARC-MOFs. Visually, PE-MOFs and ARC-MOFs are segregated into distinct regions. PE-MOFs primarily occupy the top-left quadrant of the map, suggesting that they are not extensively covered by ARC-MOFs but complement the unexplored geometric characteristics of ARC-MOFs.

**Fig. 4:**
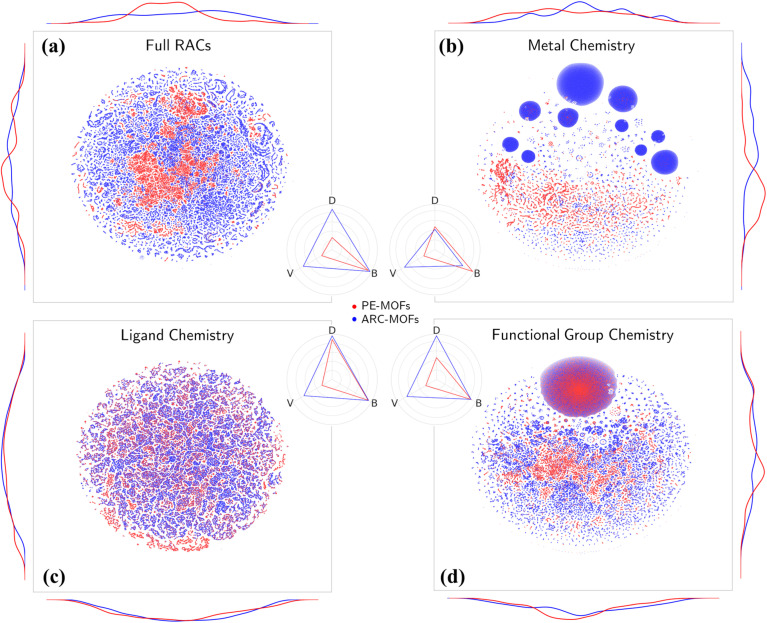
*t*-SNE maps of multi-dimensional chemical features, (a) full RACs, (b) metal chemistry, (c) ligand chemistry and (d) functional group chemistry for 94 823 PE-MOFs (red) and 279 010 ARC-MOFs (blue). The curves along the axes are feature distributions. The radar charts display diversity metrics of variety (*V*), balance (*B*) and disparity (*D*) in PE-MOFs (red) and ARC-MOFs (blue).

Chemical analysis of PE-MOFs is based on the RACs^29^ by characterizing three crucial aspects of MOF chemistry: metal, ligand, and functional group. As depicted in Fig. 4a, unlike the material space learned from geometric features (Fig. S10†), both PE-MOFs and ARC-MOFs are spread over the entire *t*-SNE map, with ARC-MOFs exhibiting a wider space. The difference is attributed to a greater structural freedom in ARC-MOFs compared with PE-MOFs, as the latter are constructed with precise or strict topological criteria. Table 1 lists the diversity metrics including variety, disparity and balance in PE-MOFs and ARC-MOFs. The variety of a database indicates the number of distinct types of structures present; the balance reflects how evenly distributed the structures are; the disparity measures how dissimilar the structures are. Apparently, PE-MOFs possess a smaller chemical space than ARC-MOFs as evidenced by a lower variety value. This likely arises from the sparse diversity of the BU reservoir^14^ exploited to construct PE-MOFs and a considerably more extensive ARC-MOF database, which is nearly three times the size of the PE-MOF database. Intriguingly, despite a limited population, a high balance value is observed in PE-MOFs (Fig. 4a–d), suggesting a well-distributed representation across different structural types. For metal chemistry and linker chemistry (Fig. 4b and c), both balance and disparity values of PE-MOFs are comparable with those of ARC-MOFs, indicating a dissimilarity-rich BU space of PE-MOFs. Although a greater diversity value in MOF banks is beneficial for computational screening and machine learning (ML),^32,52^ our approach intentionally fine-tunes exploration in the design space of MOFs. Such PE is particularly important for future RTA-based structure design employing a much wider array of BUs and becomes crucial as diffusion models have received increasingly more attention in the inverse design of MOFs.^53–55^

**Table 1:** Diversity metrics in PE-MOFs and ARC-MOFs

Feature	Database	Variety	Balance	Disparity
Full RACs	PE-MOFs	0.261	0.902	0.261
	ARC-MOFs	0.720	0.941	0.871
Metal	PE-MOFs	0.261	0.895	0.470
	ARC-MOFs	0.720	0.661	0.416
Ligand	PE-MOFs	0.261	0.920	0.885
	ARC-MOFs	0.720	0.931	0.966
Functional group	PE-MOFs	0.261	0.845	0.459
	ARC-MOFs	0.720	0.837	0.918

### 3.2. CO_2_ capture performance

**Fig. 5:**
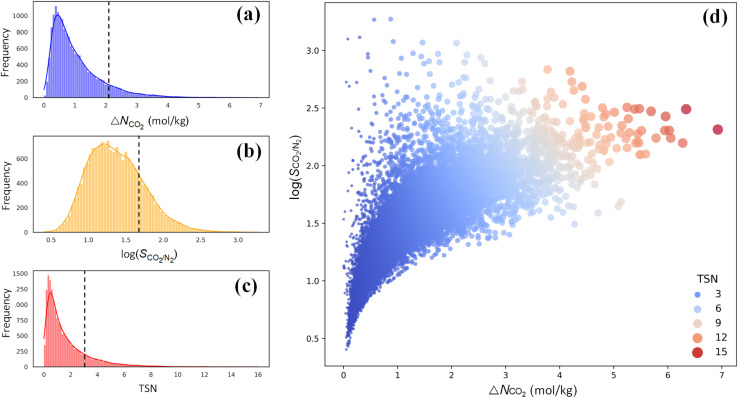
Performance of 17 173 PE-MOFs for CO_2_ capture. (a–c) Distributions of Δ*N*_CO_2__, log(*S*_CO_2_/N_2__) and TSN in 17 173 PE-MOFs. The dashed line denotes the performance of zeolite-13X as a benchmark.^38^ (d) log(*S*_CO_2_/N_2__) *versus* Δ*N*_CO_2__, with the color-coding based on TSN.

For CO_2_ capture, the performance of an adsorbent is usually quantified using three metrics: CO_2_ working capacity (Δ*N*_CO_2__), CO_2_/N_2_ selectivity *S*_CO_2_/N_2__, and a trade-off (TSN) between capacity and selectivity, TSN = log(*S*_CO_2_/N_2__) × Δ*N*_CO_2__. As illustrated in Fig. 5a–c, wide distributions are observed for Δ*N*_CO_2__, log(*S*_CO_2_/N_2__) and TSN in 17 173 PE-MOFs, specifically, in the range of 0–7 mol kg^−1^, 0.5–3.0, and 0–16, respectively. For comparison, the performance of zeolite-13X is included, which is benchmarked as a high-performing adsorbent for CO_2_ capture under dry conditions.^38^ A large number of PE-MOFs exhibit performance superior to 13X. Fig. 5d further shows log(*S*_CO_2_/N_2__) *versus* Δ*N*_CO_2__ with different TSN values. At a low Δ*N*_CO_2__, a wide range of *S*_CO_2_/N_2__ is observed approximately from 3 to 3000. With increasing Δ*N*_CO_2__, *S*_CO_2_/N_2__ generally drops and tends to approach a constant. PE-MOFs with greater TSN or superior performance are primarily clustered in the middle-right region. A total of 1207 PE-MOFs are found to surpass the TSN of 3.2 in 13X.

**Fig. 6:**
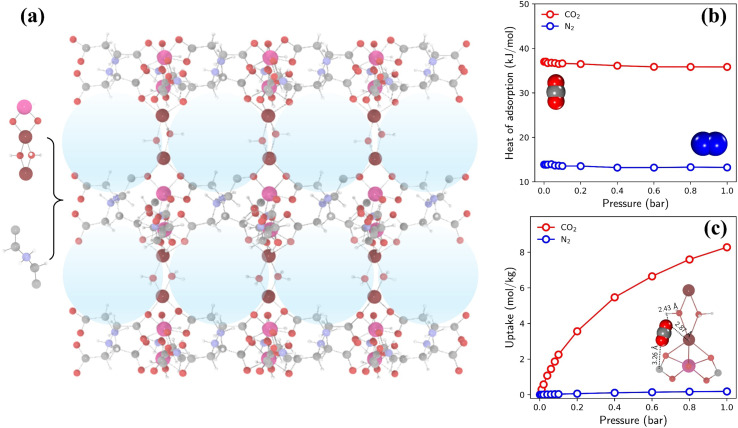
(a) Structure of a top-performing MOF (assembled from metal vertice m160 and organic edge o323) for CO_2_ capture, with blue spheres indicating the largest cavities. (b) Heats of CO_2_ and N_2_ adsorption. (c) Isotherms of CO_2_ and N_2_ adsorption, with a simulation snapshot highlighting the preferential adsorption site of CO_2_.

The highest TSN of 16.0 is observed in a PE-MOF assembled from metal vertice m160 and organic edge o323. With **bcu** topology and 8-connected metal vertices, as illustrated in Fig. 6a, this PE-MOF comprises three-dimensional pores with a diameter of 8.18 Å. Fig. 6b and c show the heats and isotherms of CO_2_ and N_2_ adsorption in this PE-MOF, calculated from GCMC simulations for a CO_2_/N_2_ mixture at 298 K and composition of 0.15/0.85. It is evident that CO_2_ adsorption significantly dominates over N_2_ across the entire pressure range, resulting in a CO_2_ adsorption capacity of 8.28 mol kg^−1^ and CO_2_/N_2_ selectivity of 346 at 1 bar. Such high CO_2_ capture performance is superior to or comparable with that of many experimentally synthesized and computationally designed MOFs reported in the literature, as compared in Table S2 and Fig. S11.†^9,15,52,56^

**Fig. 7:**
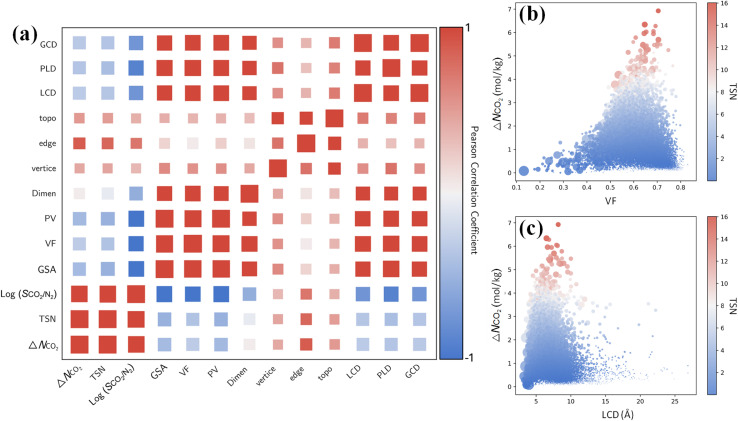
(a) Pearson correlation matrix (PCM) between CO_2_ capture performance metrics (Δ*N*_CO_2__, TSN and log(*S*_CO_2_/N_2__)) and structural characteristics (GSA, VF, PV, dimensionality, vertice, edge, topology, LCD, PLD and GCD). (b and c) Relationships of Δ*N*_CO_2__ with the VF and LCD in 17 173 MOFs, with color-coding based on TSN.

Furthermore, it is instructive to explore the interplay between CO_2_ capture performance and structure characteristics through multivariate analysis. From the Pearson correlation matrix in Fig. 7a, capture performance metrics Δ*N*_CO_2__, TSN and log(*S*_CO_2_/N_2__) exhibit negative correlations with GSA, VF, PV, LCD, PLD and GCD. These correlations are corroborated by the relationships of Δ*N*_CO_2__ with the VF and LCD in 17 173 MOFs. As shown in Fig. 7b and c, with an increasing VF or LCD, Δ*N*_CO_2__ and TSN first increase, reach maximum, and then drop; this suggests while a small pore in a MOF leads to high *S*_CO_2_/N_2__, it restricts Δ*N*_CO_2__ and TSN. The top-performing MOFs with great TSN possess VF from 0.6 to 0.7 and LCD around 7 Å. These dimensions are about one to two times the kinetic diameter of CO_2_ molecule, but less than twice that of N_2_ molecule, as also previously reported.^57^

**Fig. 8:**
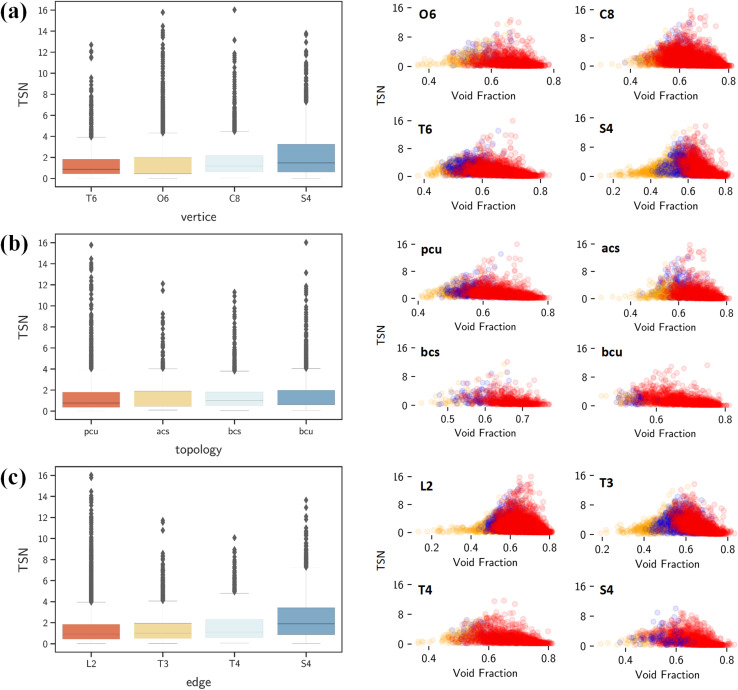
(Left) Relationships of TSN with (a) vertice, (b) topology and (c) edge. (Right) Relationships of TSN with the void fraction, and the color code indicates MOF dimensionality: 1D in orange, 2D in blue and 3D in red.

By contrast, Δ*N*_CO_2__, TSN and log(*S*_CO_2_/N_2__) show positive correlations with vertices, edges and topology as shown in Fig. 7a. This underscores the significant role of structural configuration in determining MOF performance. However, the impact may be overshadowed by the transformation of categorical variables into numerical values, which warrants deeper examination. As illustrated in Fig. 8a, MOFs with O6 and C8 vertices, fostering 3D geometries, exhibit a broad distribution of great TSN values (>8). This correlation aligns well with the finding in Fig. 8b, where 3D-MOFs with **pcu** and **bcu** topologies, characterized by larger void fractions, are densely represented among great TSN values. Regarding edges, as shown in Fig. 8c, our analysis reveals that high-performing MOFs incorporate L2 edge, which likely contributes to the formation of uniform pores; consequently, the pore space is optimized to accommodate CO_2_ molecules and enables them to align and stack efficiently.

**Fig. 9:**
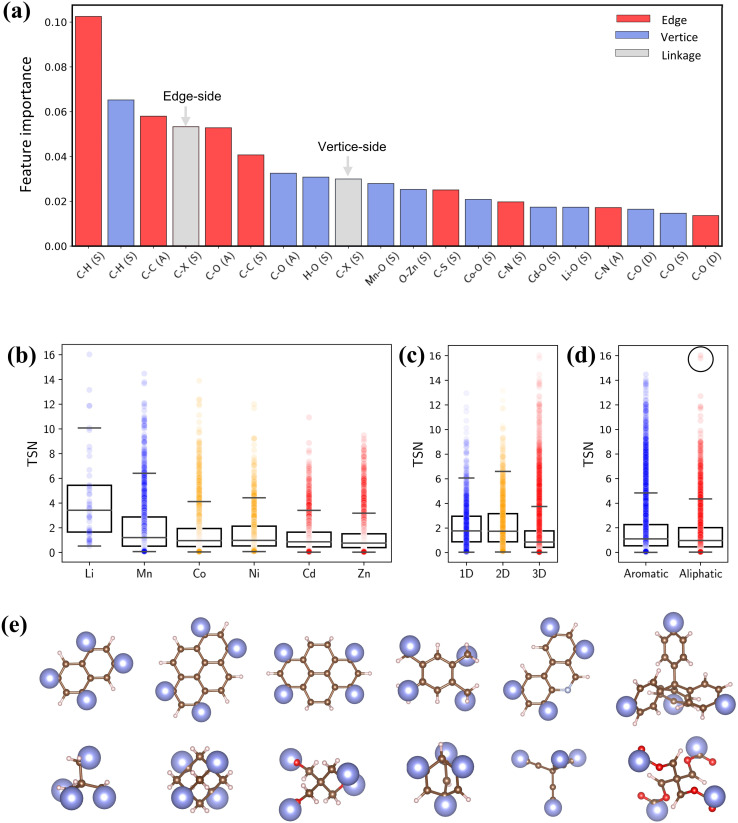
(a) SHAP interpreted feature importance of bond features. (b–d) Box plots of metal, dimensionality and linker type *versus* TSN. (e) Representative aromatic and aliphatic linkers in PE-MOFs.

To quantify the impact of chemical descriptors on CO_2_ capture, we utilized two separate Random Forest (RF) models^58^ and combined with their SHapley Additive exPlanations^59^ (SHAP) feature importance. The first model incorporates pore features along with bond types of reticulated BUs, and the second model focuses exclusively on bond types. The details of ML are provided in the ESI.† As depicted in Fig. S12,† the first model reveals that PV holds the greatest importance for CO_2_ capture. The second model shows that the presence and arrangement of carbon bonds within BUs—specifically C–H and C–C bonds—significantly affect the TSN value (Fig. 9a). These bonds typically lead to longer linkers, thereby enhancing porosity of MOFs.^60,61^ It is essential to note that the outcome of the feature importance analysis might differ depending on the dataset utilized for model training;^32^ thus, our conclusion is specific to our dataset.

Fig. 9b–d show the relationships between TSN and three key MOF characteristics (metal, dimensionality and linker type). MOFs with metals such as Li and Mn typically possess a broader range of higher TSN values compared with others. However, metals in MOFs can form as clusters or complexes and associate with various BUs.^17^ The complex configurations of these metal sites are crucial in mediating interactions (*e.g.*, dipole–dipole interactions). Regarding dimensionality, 1D and 2D MOFs, with constricted porosity as discussed previously, typically exhibit moderate TSN values due to their limited cavity space, which constrains deliverable capacity due to high surface affinity at a low pressure. In contrast, 3D MOFs, characterized by larger porosity, show significantly higher TSN values. Additionally, the edge BUs in our dataset are categorized into either aromatic or aliphatic. Aromatic linkers, with their uniform and planar geometry compared with aliphatic BUs (Fig. 9e), generally tend to exhibit higher TSN values, reflecting their capability to form MOFs with greater porosity and additional functional features. Notably, aliphatic linkers also achieve high performance in some cases (circled data points), highlighting that optimal CO_2_ capture cannot be attributed to a single chemical descriptor alone.

### 3.3. Stability and applicability of PE-MOFs

**Fig. 10:**
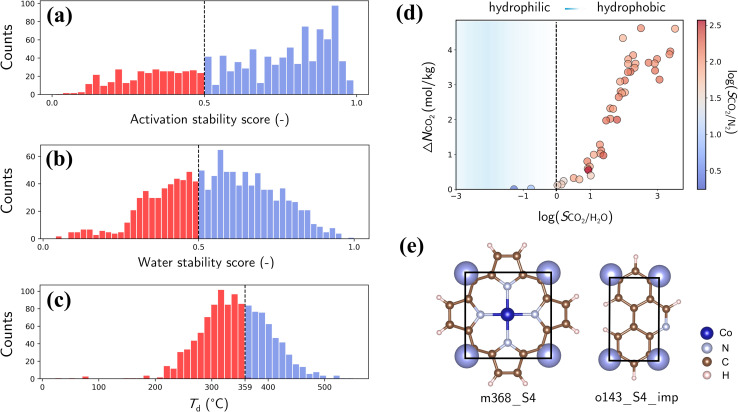
(a) Activation scores of the refined PE-MOFs (classified as collapsible if score <0.5). (b) Water stability scores of the refined PE-MOFs (classified as water unstable if score <0.5). (c) *T*_d_ of the refined PE-MOFs (359 °C used as the threshold). (d) Relationship of Δ*N*_CO_2__ with log(*S*_CO_2_/H_2_O_), with color-coding based on log(*S*_CO_2_/N_2__) (classified as hydrophobic if *S*_CO_2_/H_2_O_ > 1). (e) BUs of a top-performing MOF.

Finally, we assess the stability and applicability of the designed PE-MOFs in real-world applications, particularly for CO_2_ capture. Three critical stability metrics (activation, water and thermal)^39,40^ were used to evaluate the stability of 1207 PE-MOFs that outperform zeolite-13X for CO_2_ capture from a dry flue gas. As presented in Fig. 10a and b, the activation and water stability scores range from 0 to 1.0, categorizing PE-MOFs from unstable to stable. A threshold score of 0.5 is set to identify potentially activatable and water-stable structures. PE-MOFs with decomposition temperatures (*T*_d_) exceeding 359 °C, which is the average *T*_d_ observed in the CoRE-MOF 2019 database,^11,62^ are considered thermally stable (Fig. 10c). Among the 1207 PE-MOFs, 224 satisfy the three stability criteria. It is worthwhile to note that the ML models^39,40^ facilitate rapid stability evaluation, to a certain extent, but they have limitations. For example, the models do not consider actual activation methods such as supercritical CO_2_ drying,^63^ which can stabilize MOFs prone to collapse by conventional activation techniques. For water stability, the models do not account for interactions under varied humidity conditions.^64^ Although post-combustion CO_2_ capture typically occurs near ambient temperature, the need for thermal activation of MOFs to expel trapped solvents necessitates higher thermal stability.^65^

The 224 stable PE-MOFs were further evaluated for their CO_2_ capture from a wet flue gas (CO_2_/N_2_/H_2_O mixture at 298 K and 1 bar, with 60% relative humidity). As depicted in Fig. 10d, 34 PE-MOFs can be identified as hydrophobic (*i.e.*, *S*_CO_2_/H_2_O_ > 1) with *S*_CO_2_/N_2__ not significantly compromised. A top-performing candidate, assembled from metal vertice m368 and organic edge o143, has exceptional performance with Δ*N*_CO_2__ of 4.62 mol kg^−1^ and *S*_CO_2_/N_2__ of 121.7, even in the presence of H_2_O. It also stands out in terms of stability metrics—an activation stability score of 0.95, a water stability score of 0.59, and a *T*_d_ of 427 °C. As illustrated in Fig. 10e, this MOF possesses a planar geometry of BUs (common among other top-performers) and constricted pore apertures (4.13 × 4.83 Å) introduced by **cdm** topology, thus enhancing chemical robustness while minimizing H_2_O interference for CO_2_ capture.

## 4. Conclusions

We have developed a fine-tuned RTA for structure prediction toward the digital discovery of MOFs. By addressing combinatorial explosion and effectively navigating a large array of BUs, our approach enhances the efficiency and precision to assemble BUs. A total of 94 823 PE-MOFs are designed with a more variety of geometries compared with ARC-MOFs despite a relatively smaller chemical space. For CO_2_ capture, the performance of PE-MOFs exhibits negative correlations with several geometric features (*i.e.*, GSA, VF, PV, LCD, PLD and GCD), but positive correlations with structural configuration (*i.e.*, vertices, edges and topology). Further quantitative insights are probed with machine learning models, highlighting the indispensable role of pore volume and the configuration of carbon bonds in determining CO_2_ capture performance. A larger number of PE-MOFs are found to be superior to benchmarked zeolite-13X. By integrating three stability metrics (activation, water and thermal), stable PE-MOFs are identified with high-performance for CO_2_ capture from a wet flue gas.

The strategic assembly of BUs based on geometric signatures and topological compatibility not only mitigates combinatorial explosion by filtering out infeasible topologies, but also demonstrates the potential of digital chemistry in discovering high-performing materials. Our approach highlights the transformative impact of synergizing precision engineering with digital reticular chemistry, and it would advance further innovation in the intelligent design of porous crystals beyond MOFs. In future research, we will focus on expanding the diversity of BUs, particularly by customizing property-triggered BUs and integrating mechanical-, water- and thermal-stability, and other important stability measures. Advanced machine learning techniques will be incorporated to streamline the design process and enhance practical applicability.

## Data availability

Data and codes related to this study are available on GitHub https://github.com/xiaoyu961031/Fine-tuned-RTA. All computationally predicted structures are available on Zenodo https://zenodo.org/records/11480898.

## Author contributions

XW conceptualized the project, developed the approach and wrote the manuscript. All authors reviewed and edited the manuscript. JJ acquired the funding and supervised the project.

## Conflicts of interest

There is no conflict of interests to declare.

## Supplementary Material

SC-OLF-D4SC05616G-s001

## References

[cit1] Li H., Eddaoudi M., O'Keeffe M., Yaghi O. M. (1999). Design and synthesis of an exceptionally stable and highly porous metal-organic framework. Nature.

[cit2] Furukawa H., Cordova K. E., O’Keeffe M., Yaghi O. M. (2013). The chemistry and applications of metal-organic frameworks. Science.

[cit3] Lyu H., Ji Z., Wuttke S., Yaghi O. M. (2020). Digital Reticular Chemistry. Chem.

[cit4] Draznieks C. M., Newsam J. M., Gorman A. M., Freeman C. M., Férey G. (2000). De Novo Prediction of Inorganic Structures Developed through Automated Assembly of Secondary Building Units (AASBU Method). Angew. Chem., Int. Ed..

[cit5] Darby J. P., Arhangelskis M., Katsenis A. D., Marrett J. M., Friščić T., Morris A. J. (2020). Ab Initio Prediction of Metal-Organic Framework Structures. Chem. Mater..

[cit6] Xu Y., Marrett J. M., Titi H. M., Darby J. P., Morris A. J., Friščić T., Arhangelskis M. (2023). Experimentally Validated Ab Initio Crystal Structure Prediction of Novel Metal–Organic Framework Materials. J. Am. Chem. Soc..

[cit7] Wilmer C. E., Leaf M., Lee C. Y., Farha O. K., Hauser B. G., Hupp J. T., Snurr R. Q., Wilmer C. E., Leaf M., Lee C. Y. (2011). *et al.*, Large-scale screening of hypothetical metal–organic frameworks. Nat. Chem..

[cit8] Keupp J., Keupp J., Schmid R., Schmid R. (2018). TopoFF: MOF structure prediction using specifically optimized blueprints. Faraday Discuss..

[cit9] Boyd P. G., Chidambaram A., García-Díez E., Ireland C. P., Daff T. D., Bounds R., Gładysiak A., Schouwink P., Moosavi S. M., Maroto-Valer M. M. (2019). *et al.*, Data-driven design of metal–organic frameworks for wet flue gas CO_2_ capture. Nature.

[cit10] Lee S., Kim B., Cho H., Lee H., Lee S. Y., Cho E. S., Kim J. (2021). Computational Screening of Trillions of Metal-Organic Frameworks for High-Performance Methane Storage. ACS Appl. Mater. Interfaces.

[cit11] Nandy A., Yue S., Oh C., Duan C., Terrones G. G., Chung Y. G., Kulik H. J. (2023). A database of ultrastable MOFs reassembled from stable fragments with machine learning models. Matter.

[cit12] Jiang J. (2019). Computational screening of metal–organic frameworks for CO_2_ separation. Curr. Opin. Green Sustainable Chem..

[cit13] Rosen A. S., Iyer S. M., Ray D., Yao Z., Aspuru-Guzik A., Gagliardi L., Notestein J. M., Snurr R. Q. (2021). Machine learning the quantum-chemical properties of metal–organic frameworks for accelerated materials discovery. Matter.

[cit14] Burner J., Luo J., White A., Mirmiran A., Kwon O., Boyd P. G., Maley S., Gibaldi M., Simrod S., Ogden V., Woo T. K. (2023). ARC-MOF: A Diverse Database of Metal-Organic Frameworks with DFT-Derived Partial Atomic Charges and Descriptors for Machine Learning. Chem. Mater..

[cit15] Mohamed S. A., Zhao D., Jiang J. (2023). Integrating stability metrics with high-throughput computational screening of metal–organic frameworks for CO_2_ capture. Commun. Mater..

[cit16] Yaghi O. M. (2019). Reticular Chemistry in All Dimensions. ACS Cent. Sci..

[cit17] Kalmutzki M. J., Hanikel N., Yaghi O. M. (2018). Secondary building units as the turning point in the development of the reticular chemistry of MOFs. Sci. Adv..

[cit18] Gibaldi M., Kwon O., White A., Burner J., Woo T. K. (2022). The HEALED SBU Library of Chemically Realistic Building Blocks for Construction of Hypothetical Metal-Organic Frameworks. ACS Appl. Mater. Interfaces.

[cit19] Jablonka K. M., Rosen A. S., Krishnapriyan A. S., Smit B. (2023). An Ecosystem for Digital Reticular Chemistry. ACS Cent. Sci..

[cit20] Padial N. M., Procopio E. Q., Montoro C., López E., Oltra J. E., Colombo V., Maspero A., Masciocchi N., Galli S., Senkovska I. (2013). *et al.*, Highly Hydrophobic Isoreticular Porous Metal–Organic Frameworks for the Capture of Harmful Volatile Organic Compounds. Angew. Chem..

[cit21] Gómez-Gualdrón D. A., Colón Y. J., Zhang X., Wang T. C., Chen Y.-S., Hupp J. T., Yildirim T., Farha O. K., Zhang J., Snurr R. Q. (2016). Evaluating topologically diverse metal–organic frameworks for cryo-adsorbed hydrogen storage. Energy Environ. Sci..

[cit22] Boyd P. G., Woo T. K. (2016). A generalized method for constructing hypothetical nanoporous materials of any net topology from graph theory. CrystEngComm.

[cit23] Chui S. S. Y., Lo S. M. F., Charmant J. P. H., Orpen A. G., Williams I. D. (1999). A chemically functionalizable nanoporous material [Cu_3_(TMA)_2_(H_2_O)_3_]_(n)_. Science.

[cit24] O'Keeffe M., Peskov M. A., Ramsden S. J., Yaghi O. M. (2008). The Reticular Chemistry Structure Resource (RCSR) database of, and symbols for, crystal nets. Acc. Chem. Res..

[cit25] Mercado R., Fu R. S., Yakutovich A. V., Talirz L., Haranczyk M., Smit B. (2018). In Silico Design of 2D and 3D Covalent Organic Frameworks for Methane Storage Applications. Chem. Mater..

[cit26] Rappé A. K., Casewit C. J., Colwell K. S., Goddard W. A., Skiff W. M. (1992). UFF, a Full Periodic Table Force Field for Molecular Mechanics and Molecular Dynamics Simulations. J. Am. Chem. Soc..

[cit27] Kancharlapalli S., Gopalan A., Haranczyk M., Snurr R. Q. (2021). Fast and Accurate Machine Learning Strategy for Calculating Partial Atomic Charges in Metal-Organic Frameworks. J. Chem. Theory Comput..

[cit28] Willems T. F., Rycroft C. H., Kazi M., Meza J. C., Haranczyk M. (2012). Algorithms and tools for high-throughput geometry-based analysis of crystalline porous materials. Microporous Mesoporous Mater..

[cit29] Janet J. P., Kulik H. J. (2017). Resolving Transition Metal Chemical Space: Feature Selection for Machine Learning and Structure-Property Relationships. J. Phys. Chem. A.

[cit30] Nandy A., Duan C., Janet J. P., Gugler S., Kulik H. J. (2018). Strategies and Software for Machine Learning Accelerated Discovery in Transition Metal Chemistry. Ind. Eng. Chem. Res..

[cit31] Nandy A., Duan C., Taylor M. G., Liu F., Steeves A. H., Kulik H. J. (2021). Computational Discovery of Transition-metal Complexes: From High-throughput Screening to Machine Learning. Chem. Rev..

[cit32] Moosavi S. M., Nandy A., Jablonka K. M., Ongari D., Janet J. P., Boyd P. G., Lee Y., Smit B., Kulik H. J. (2020). Understanding the diversity of the metal-organic framework ecosystem. Nat. Commun..

[cit33] Lee J., Lee I., Park J., Kim H., Kim M., Min K., Lee S. (2023). Optimal Surrogate Models for Predicting the Elastic Moduli of Metal–Organic Frameworks via Multiscale Features. Chem. Mater..

[cit34] Tang H., Xu Q., Wang M., Jiang J. (2021). Rapid screening of metal–organic frameworks for propane/propylene separation by synergizing molecular simulation and machine learning. ACS Appl. Mater. Interfaces.

[cit35] Zhang Z., Tang H., Wang M., Lyu B., Jiang Z., Jiang J. (2023). Metal–Organic Frameworks for Water Harvesting: Machine Learning-Based Prediction and Rapid Screening. ACS Sustain. Chem. Eng..

[cit36] Ioannidis E. I., Gani T. Z. H., Kulik H. J. (2016). molSimplify: a toolkit for automating discovery in inorganic chemistry. J. Comput. Chem..

[cit37] Van Der Maaten L., Hinton G. (2008). Visualizing Data using t-SNE. J. Mach. Learn. Res..

[cit38] Ho M. T., Allinson G. W., Wiley D. E. (2008). Reducing the Cost of CO_2_ Capture from Flue Gases Using Pressure Swing Adsorption. Ind. Eng. Chem. Res..

[cit39] Nandy A., Terrones G., Arunachalam N., Duan C., Kastner D. W., Kulik H. J. (2022). MOFSimplify, machine learning models with extracted stability data of three thousand metal–organic frameworks. Sci. Data.

[cit40] Terrones G. G., Huang S.-P., Rivera M. P., Yue S., Hernandez A., Kulik H. J. (2024). Metal–Organic Framework Stability in Water and Harsh Environments from Data-Driven Models Trained on the Diverse WS24 Data Set. J. Am. Chem. Soc..

[cit41] Potoff J. J., Siepmann J. I. (2001). Vapor–liquid equilibria of mixtures containing alkanes, carbon dioxide, and nitrogen. AIChE J..

[cit42] Vorholz J., Harismiadis V. I., Rumpf B., Panagiotopoulos A. Z., Maurer G. (2000). Vapor+liquid equilibrium of water, carbon dioxide, and the binary system, water+carbon dioxide, from molecular simulation. Fluid Phase Equilib..

[cit43] Mayo S. L., Olafson B. D., Goddard W. A. (1990). DREIDING: a generic force field for molecular simulations. J. Phys. Chem..

[cit44] Manz T. A., Sholl D. S. (2010). Chemically Meaningful Atomic Charges That Reproduce the Electrostatic Potential in Periodic and Nonperiodic Materials. J. Chem. Theory Comput..

[cit45] Limas N. G., Manz T. A. (2018). Introducing DDEC6 atomic population analysis: part 4. Efficient parallel computation of net atomic charges, atomic spin moments, bond orders, and more. RSC Adv..

[cit46] Kresse G. (1995). Ab initio molecular dynamics for liquid metals. J. Non-Cryst. Solids.

[cit47] Kresse G., Furthmüller J. (1996). Efficient iterative schemes for ab initio total-energy calculations using a plane-wave basis set. Phys. Rev. B: Condens. Matter Mater. Phys..

[cit48] Perdew J. P., Burke K., Ernzerhof M. (1996). Generalized Gradient Approximation Made Simple. Phys. Rev. Lett..

[cit49] Grimme S., Antony J., Ehrlich S., Krieg H. (2010). A consistent and accurate ab initio parametrization of density functional dispersion correction (DFT-D) for the 94 elements H-Pu. J. Chem. Phys..

[cit50] Grimme S., Ehrlich S., Goerigk L. (2011). Effect of the damping function in dispersion corrected density functional theory. J. Comput. Chem..

[cit51] Dubbeldam D., Calero S., Ellis D. E., Snurr R. Q. (2016). RASPA: molecular simulation software for adsorption and diffusion in flexible nanoporous materials. Mol. Simul..

[cit52] Majumdar S., Moosavi S. M., Jablonka K. M., Ongari D., Smit B. (2021). Diversifying Databases of Metal Organic Frameworks for High-Throughput Computational Screening. ACS Appl. Mater. Interfaces.

[cit53] Fu X. , Xie T., Rosen A. S., Jaakkola T. and Smith J., MOFDiff: Coarse-grained Diffusion for Metal-Organic Framework Design, arXiv, 2023, preprint, arXiv:2310.10732, 10.48550/arXiv.2310.10732

[cit54] Xiao H., Li R., Shi X., Chen Y., Zhu L., Chen X., Wang L. (2023). An invertible, invariant crystal representation for inverse design of solid-state materials using generative deep learning. Nat. Commun..

[cit55] Park H., Yan X., Zhu R., Huerta E. A., Chaudhuri S., Cooper D., Foster I., Tajkhorshid E. (2024). A generative artificial intelligence framework based on a molecular diffusion model for the design of metal-organic frameworks for carbon capture. Commun. Chem..

[cit56] Lin J. B., Nguyen T. T. T., Vaidhyanathan R., Burner J., Taylor J. M., Durekova H., Akhtar F., Mah R. K., Ghaffari-Nik O., Marx S. (2021). *et al.*, A scalable metal-organic framework as a durable physisorbent for carbon dioxide capture. Science.

[cit57] Cao X., Wang Z., Qiao Z., Zhao S., Wang J. (2019). Penetrated COF Channels: Amino Environment and Suitable Size for CO_2_ Preferential Adsorption and Transport in Mixed Matrix Membranes. ACS Appl. Mater. Interfaces.

[cit58] Ho T. K. , Random decision forests, IEEE, Montréal, Canada, 1995

[cit59] Lundberg S. M. and Lee S.-I., A unified approach to interpreting model predictions, in NIPS'17, Long Beach, California, USA, 2017

[cit60] Bailey T., Jackson A., Berbece R.-A., Wu K., Hondow N., Martin E. (2023). Gradient Boosted Machine Learning Model to Predict H_2_, CH_4_, and CO_2_ Uptake in Metal–Organic Frameworks Using Experimental Data. J. Chem. Inf. Model..

[cit61] Wang R., Meng Q., Zhang L., Wang H., Dai F., Guo W., Zhao L., Sun D. (2014). Investigation of the effect of pore size on gas uptake in two fsc metal–organic frameworks. Chem. Commun..

[cit62] Chung Y. G., Haldoupis E., Bucior B. J., Haranczyk M., Lee S., Zhang H., Vogiatzis K. D., Milisavljevic M., Ling S., Camp J. S. (2019). *et al.*, Advances, Updates, and Analytics for the Computation-Ready, Experimental Metal–Organic Framework Database: CoRE MOF 2019. J. Chem. Eng. Data.

[cit63] Frimpong D. M., Baojian L. (2019). Recent Advances of Supercritical CO_2_ in Green Synthesis and Activation of Metal–Organic Frameworks. J. Inorg. Organomet. Polym. Mater..

[cit64] Burtch N. C., Jasuja H., Walton K. S. (2014). Water Stability and Adsorption in Metal–Organic Frameworks. Chem. Rev..

[cit65] Woodliffe J. L., Ferrari R. S., Ahmed I., Laybourn A. (2021). Evaluating the purification and activation of metal-organic frameworks from a technical and circular economy perspective. Coord. Chem. Rev..

