# Supplementary material for: Precision-engineered metal–organic frameworks: fine-tuning reverse topological structure prediction and design
Source: Chem Sci. 2024 Sep 25;15(40):16467–79. doi: 10.1039/d4sc05616g (PMC11423560; doi:10.1039/d4sc05616g)
Supplement: SC-015-D4SC05616G-s001 [file SC-015-D4SC05616G-s001.pdf]

Supplementary Information (SI) for Chemical Science.
This journal is © The Royal Society of Chemistry 2024

Electronic supplementary information

Precision-engineered metal–organic frameworks: fine-tuning reverse topological structure prediction and design

Xiaoyu Wu and Jianwen Jiang*

Department of Chemical and Bimolecular Engineering, National University of Singapore, 117576, Singapore

Table of Contents

1. **Geometric prototypes** S2
2. **Topological constraints** S4
3. **Fine-tuned reverse topological approach** S6
4. **Force field parameters** S9
5. **Comparison of charge estimations** S11
6. **Topology distribution and its impact on pore features** S12
7. **t-SNE map of geometric features** S14
8. **Comparative performance analysis of top-performing PE-MOF** S15
9. **Machine learning for CO_2 capture in PE-MOFs** S16

1. Geometric prototypes

```
## python##
import numpy as np

O6 = np.array([
    [1, 0, 0], [0, 1, 0], [0, 0, 1],
    [-1, 0, 0], [0, -1, 0], [0, 0, -1]
])

T6 = np.array([
    [np.sqrt(3)/2, 0.5, 0.5],
    [-np.sqrt(3)/2, 0.5, 0.5],
    [0, -1, 0.5],
    [np.sqrt(3)/2, 0.5, -0.5],
    [-np.sqrt(3)/2, 0.5, -0.5],
    [0, -1, -0.5]
])

C12 = np.array([
    [1, 0, 1], [-1, 0, 1], [1, 0, -1], [-1, 0, -1],
    [0, 1, 1], [0, -1, 1], [0, 1, -1], [0, -1, -1],
    [1, 1, 0], [-1, 1, 0], [1, -1, 0], [-1, -1, 0]
])

I12 = np.array([
    [-1, (1 + np.sqrt(5))/2, 0], [1, (1 + np.sqrt(5))/2, 0],
    [-1, -(1 + np.sqrt(5))/2, 0], [1, -(1 + np.sqrt(5))/2, 0],
    [0, -1, (1 + np.sqrt(5))/2], [0, 1, (1 + np.sqrt(5))/2],
    [0, -1, -(1 + np.sqrt(5))/2], [0, 1, -(1 + np.sqrt(5))/2],
    [(1 + np.sqrt(5))/2, 0, -1], [(1 + np.sqrt(5))/2, 0, 1],
    [-(1 + np.sqrt(5))/2, 0, -1], [-(1 + np.sqrt(5))/2, 0, 1]
])

H12 = np.array([
    [np.cos(theta), np.sin(theta), 0.5] for theta in np.linspace(0, 2*np.pi, 6, endpoint=False)
] + [
    [np.cos(theta), np.sin(theta), -0.5] for theta in np.linspace(0, 2*np.pi, 6, endpoint=False)
])

T12 = np.array([
    [1, 1, 1], [-1, 1, 1], [1, -1, 1], [-1, -1, 1],
    [0, 1, -np.sqrt(2)], [0, -1, -np.sqrt(2)],
    [1, np.sqrt(2), 0], [-1, np.sqrt(2), 0],
    [-np.sqrt(2), 0, 1], [np.sqrt(2), 0, -1],
    [1, -np.sqrt(2), 0], [-1, -np.sqrt(2), 0]
```

])

O6 = np.array([[1, 0, 0], [0, 1, 0], [0, 0, 1], [-1, 0, 0], [0, -1, 0], [0, 0, -1]])

T6 = np.array([[np.sqrt(3)/2, 0.5, 0.5], [-np.sqrt(3)/2, 0.5, 0.5], [0, -1, 0.5], [np.sqrt(3)/2, 0.5, -0.5], [-np.sqrt(3)/2, 0.5, -0.5], [0, -1, -0.5]

C12 = np.array([[1, 0, 1], [-1, 0, 1], [1, 0, -1], [-1, 0, -1], [0, 1, 1], [0, -1, 1], [0, 1, -1], [0, -1, -1], [1, 1, 0], [-1, 1, 0], [1, -1, 0], [-1, -1, 0]])

I12 = np.array([[-1, (1 + np.sqrt(5))/2, 0], [1, (1 + np.sqrt(5))/2, 0], [-1, -(1 + np.sqrt(5))/2, 0], [1, -(1 + np.sqrt(5))/2, 0], [0, -1, (1 + np.sqrt(5))/2], [0, 1, (1 + np.sqrt(5))/2], [0, -1, -(1 + np.sqrt(5))/2], [0, 1, -(1 + np.sqrt(5))/2], [(1 + np.sqrt(5))/2, 0, -1], [(1 + np.sqrt(5))/2, 0, 1], [-(1 + np.sqrt(5))/2, 0, -1], [-(1 + np.sqrt(5))/2, 0, 1]])

H12 = np.array([[np.cos(theta), np.sin(theta), 0.5] for theta in np.linspace(0, 2*np.pi, 6, endpoint=False)] + [[np.cos(theta), np.sin(theta), -0.5] for theta in np.linspace(0, 2*np.pi, 6, endpoint=False)])

T12 = np.array([[1, 1, 1], [-1, 1, 1], [1, -1, 1], [-1, -1, 1], [0, 1, -np.sqrt(2)], [0, -1, -np.sqrt(2)], [1, np.sqrt(2), 0], [-1, np.sqrt(2), 0], [-np.sqrt(2), 0, 1], [np.sqrt(2), 0, -1], [1, -np.sqrt(2), 0], [-1, -np.sqrt(2), 0]])

##python##

2. Topological constraints

##python##
Topological constraints = {
("T3", "L2"): ["**srs**"],
("T3", "T3"): ["**bwt**", "**pyo**"],
("T3", "S4"): ['**fjh**', '**fmj**', '**gee**', '**iab**'],
("T3", "T4"): ['**ofp**'],
("T3", "O6"): ["**anh**", "**ant**", "**apo**", "**brk**", "**cml**", "**eea**", "**qom**", "**rtl**", "**tsx**", "**zzz**"],
("S4", "L2"): ["**nbo**", "**lvt**", "**rhr**"],
("S4", "T3"): ["**pto**", "**tbo**"],
("S4", "S4"): ["**cdl**", "**cdm**", "**cdn**", "**cds**", "**cdz**", "**mot**", "**muo**", "**qdl**", "**qzd**", "**ssd**", "**sse**", "**ssf**", "**sst**"],
("S4", "T4"): ["**pts**"],
("S4", "O6"): ["**myd**", "**ybh**"],
("T4", "L2"): ["**dia**", "**lcs**", "**qtz**", "**sod**"],
("T4", "T3"): ["**bor**", "**ctn**"],
("T4", "S4"): ["**fgl**", "**mog**", "**pds**", "**pth**", "**pti**", "**ptr**", "**ptt**"],
("T4", "T4"): ["**bn**l", "**byl**", "**cag**", "**cbt**", "**coe**", "**crb**", "**fel**", "**icm**", "**kea**", "**lon**", "**pcl**", "**sca**", '**tpd**', "**ucn**"],
("T4", "O6"): ["**alw**", "**bix**", "**cor**", "**spl**", "**toc**"],
("H6", "L2"): ["**hxg**"],
("H6", "S4"): ["**she**"],
("O6", "L2"): ["**pcu**", "**bcs**", "**crs**", "**reo**"],
("O6", "T3"): ["**pyr**", "**spn**"],
("O6", "S4"): ["**soc**"],
("O6", "T4"): ["**gar**", "**iac**", "**ibd**", "**toc**"],
("T6", "L2"): ["**lcy**", "**acs**"],
("T6", "T3"): ["**dag**", "**hwx**", "**moo**", "**sab**", "**sit**", "**ydq**"],
("T6", "S4"): ["**stp**"],
("T6", "T4"): ["**fsi**", "**hea**", "**tpt**"],
("T6", "H6"): ["**htp**"],
("T6", "O6"): ["**nia**"],
("C8", "L2"): ["**bcu**"],
("C8", "T3"): ["**the**"],
("C8", "S4"): ["**scu**", "**csq**", "**sqc**"],
("C8", "T4"): ['**flu**'],
("C8", "O6"): ["**ocu**"],
("C12", "L2"): ["**fcu**"],
("C12", "S4"): ["**ftw**"],
("C12", "T4"): ["**edc**"],
("I12", "T4"): ["**ith**"],
("H12", "T3"): ["**aea**"],

```
    ("H12", "S4"): ["shp"],
    ("T12", "T3"): ["ttt"],
    ("T12", "H6"): ["mgc"],
    ("C24", "T4"): ["twf"],
    }
##python
```

3. Fine-tuned reverse topological approach

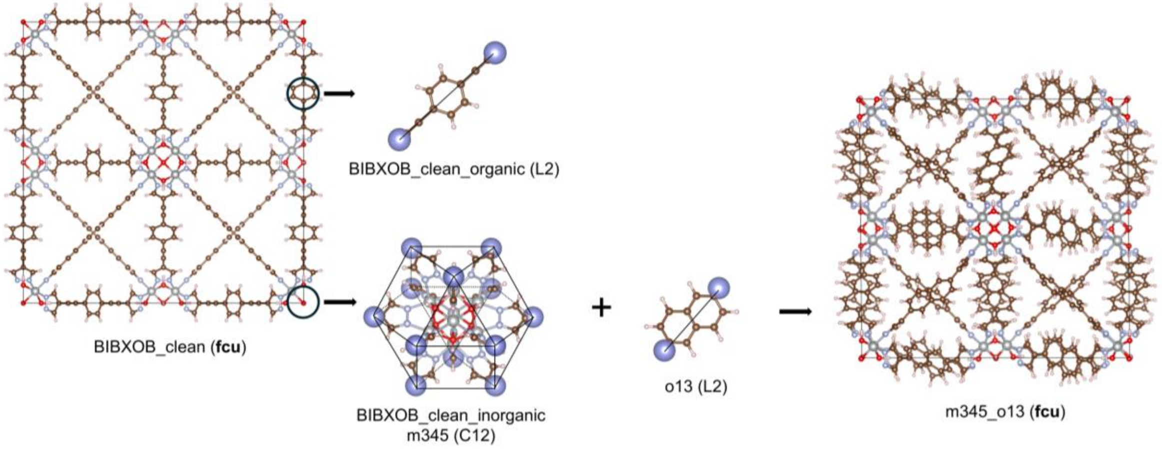

Fig. S1. Assembly of m345 from BIBXOB_clean[1] combined with o13 from HEALED database.[2]

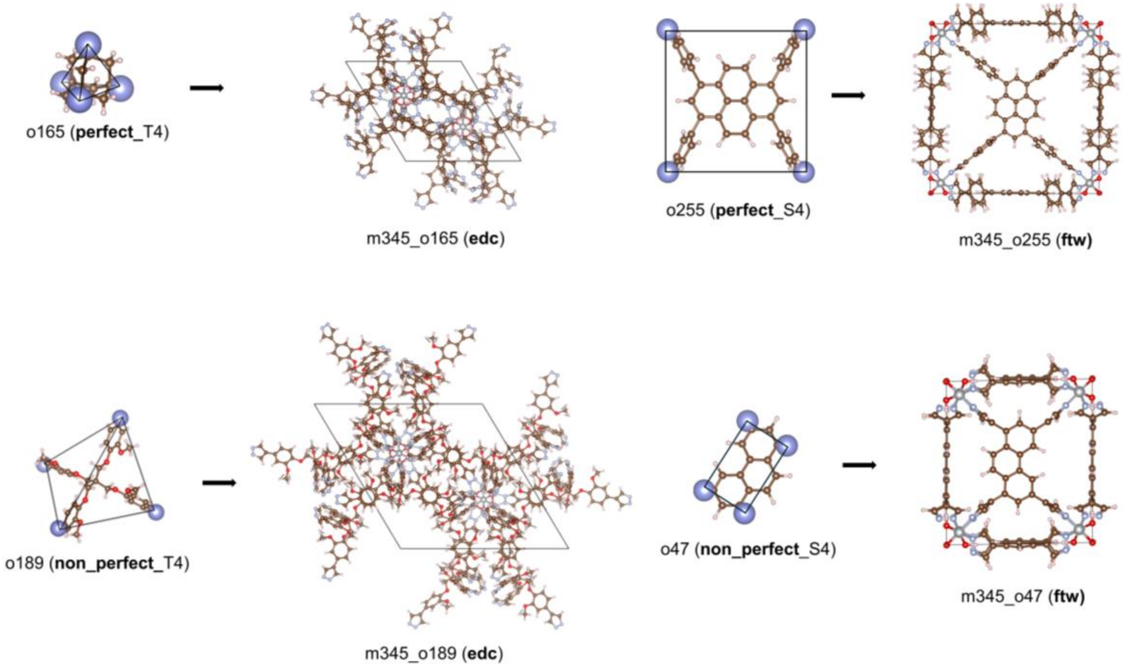

Fig. S2. Assembly variations of m345 with different edge BUs demonstrating topological flexibility in MOF construction.

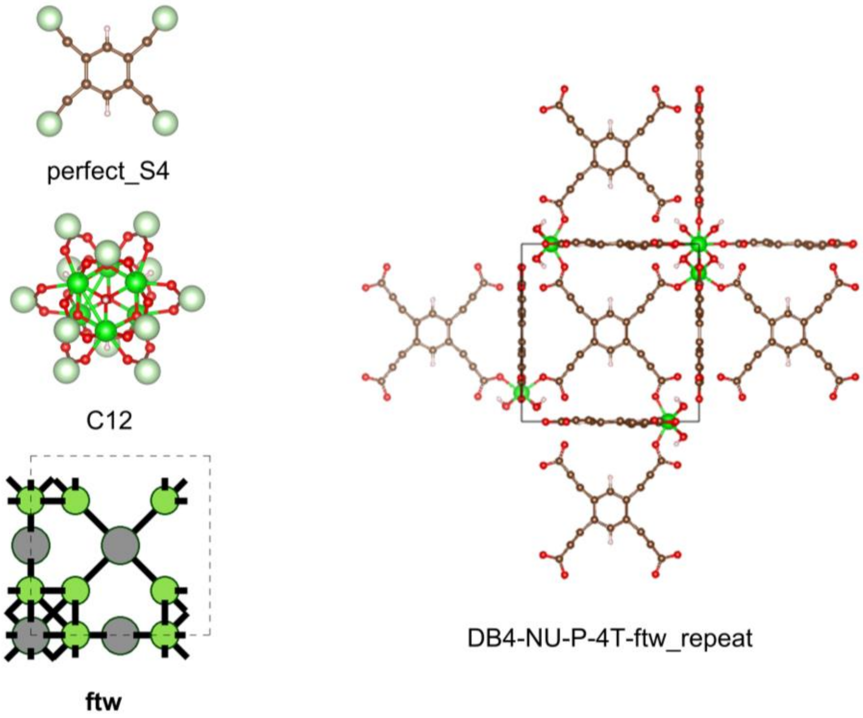

Fig. S3. Idealized BU assembly in DB4-NU-P-4T-ftw_repeat from the ARC-MOF database[3] and Gómez Gauldrón et al.'s work.[4]

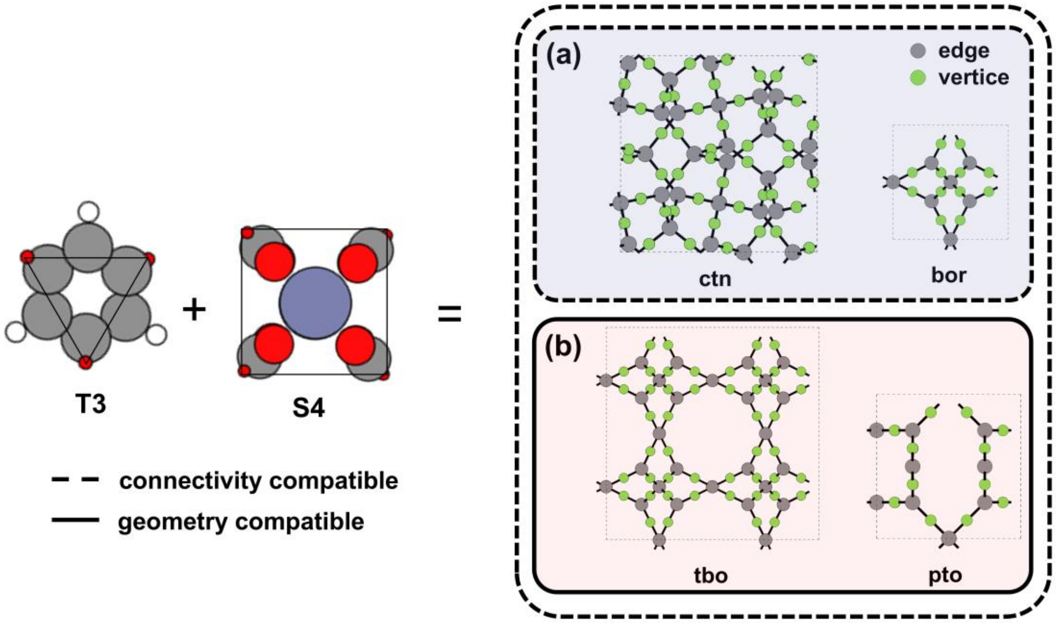

Fig. S4. Compatibility analysis of BUs across multiple topologies. Topologies in both (a) and (b) satisfy connectivity compatibility (dashed line); only topologies in (b) satisfy geometry compatibility (solid line).

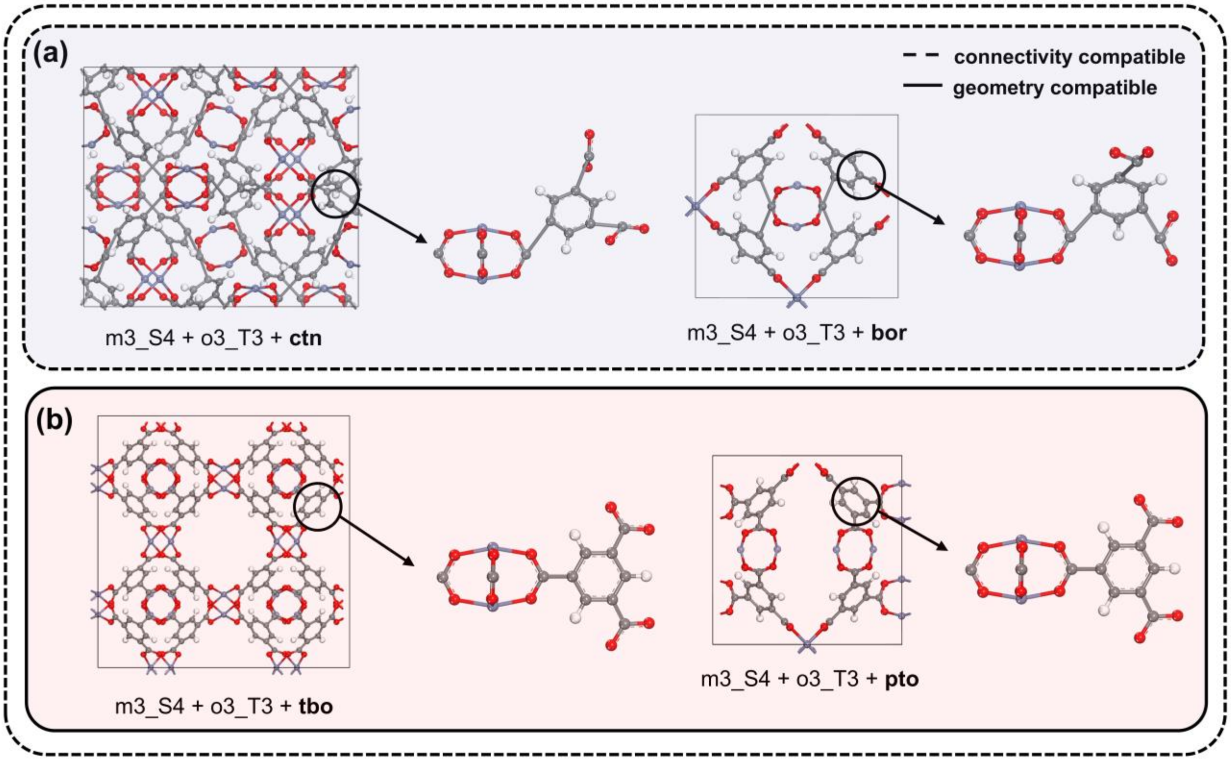

Fig. S5. Compatibility analysis of BUs across four topologies, demonstrating practical and favorable configurations based on geometry compatibility compared to connectivity compatibility. Topologies in both (a) and (b) satisfy connectivity compatibility (dashed line); only topologies in (b) satisfy geometry compatibility (solid line).

4. Force field parameters

Table S1. Force field parameters from TraPPE,[5] DREIDING[6] and UFF.[7]

Atom type	ε/k_B (K)	σ (Å)
**C_CO$_2$**	27.0	2.80
**O_CO$_2$**	79.0	3.05
**N_N$_2$**	36.0	3.31
**com_N$_2$**	0	0
**Ag**	18.11	2.80
**Al**	254.09	4.01
**B**	47.8085	3.5814
**Ba**	183.15	3.3
**Be**	42.77	2.45
**Br**	186.2016	3.51905
**C**	47.888	3.473
**Ca**	119.75	3.03
**Cd**	114.72	2.54
**Ce**	6.54	3.17
**Cl**	142.5700	3.51932
**Co**	7.04	2.56
**Cr**	7.55	2.69
**Cu**	2.52	3.11
**Dy**	3.52	3.05
**E-**	3.52	3.02
**F**	36.4854	3.0932
**Fe**	6.54	2.59
**Ga**	208.81	3.9
**Gd**	4.53	3
**H**	7.649	2.846
**Hf**	36.23	2.8
**Ho**	3.52	3.04
**I**	170.57	4.01
**In**	301.39	3.98
**Ir**	36.73	2.53
**K**	17.61	3.4
**La**	8.55	3.14
**Li**	12.58	2.18

**Lu**	20.63	3.24
**Mg**	55.85	2.69
**Mn**	6.54	2.64
**Mo**	28.18	2.72
**N**	38.975	3.2626
**Na**	15.09	2.66
**Nd**	5.03	3.18
**Ni**	7.55	2.52
**Np**	9.56	3.05
**O**	48.190	3.0332
**P**	161.1392	3.6972
**Pb**	333.59	3.83
**Pr**	5.03	3.21
**Pt**	40.25	2.45
**Rh**	26.67	2.61
**S**	173.1172	3.5903
**Sc**	9.56	2.94
**Se**	146.42	3.75
**Si**	202.27	3.83
**Sm**	4.03	3.14
**Sr**	118.24	3.24
**Tm**	3.02	3.01
**U**	11.07	3.02
**V**	8.05	2.8
**W**	33.71	2.73
**Y**	36.23	2.98
**Yb**	114.72	2.99
**Zn**	62.39	2.46

5. Comparison of charge estimations

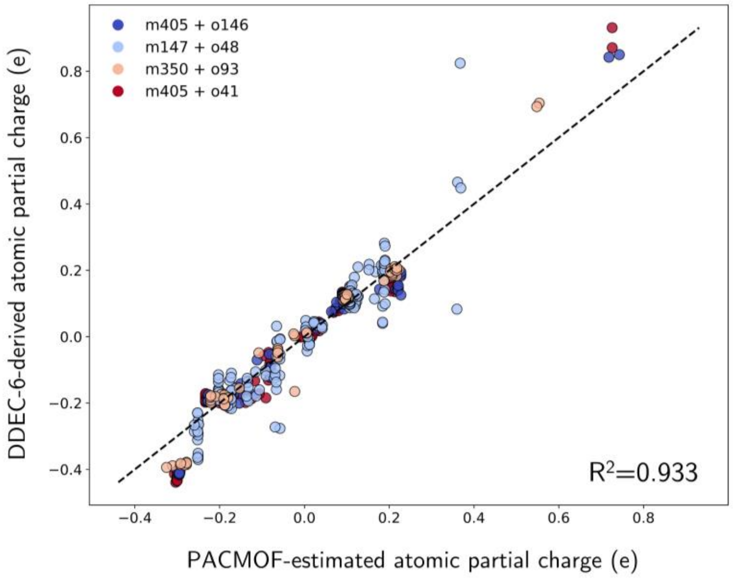

Fig. S6. Comparison of PACMOF charges and DDEC charges in four PE-MOFs.

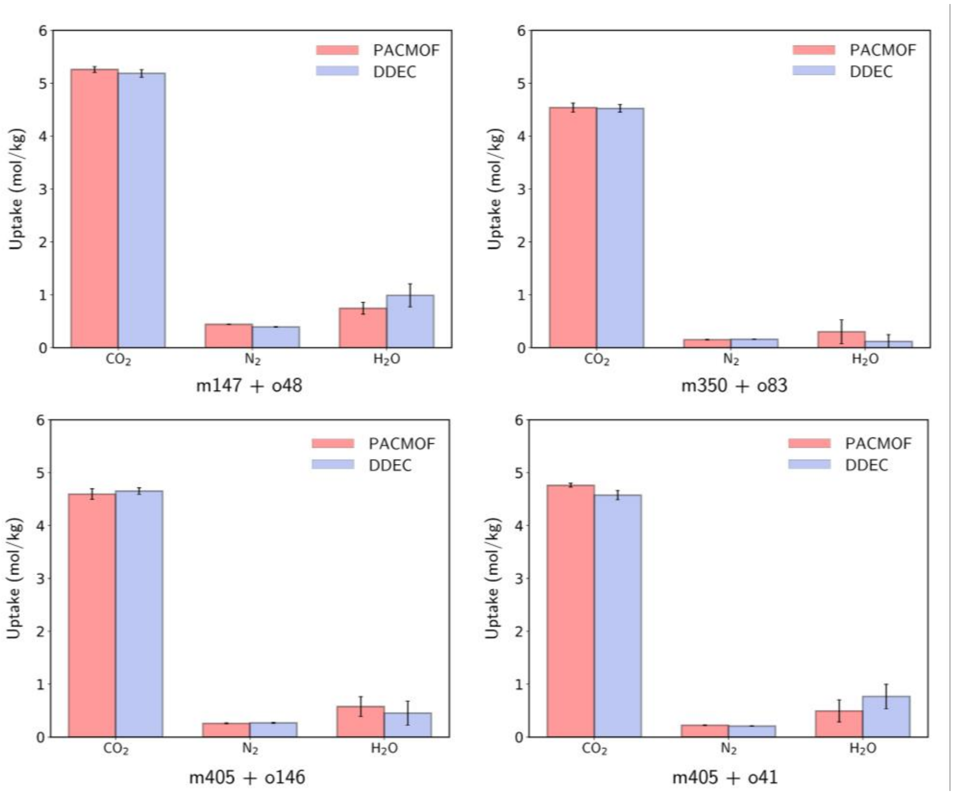

Fig. S7. CO_2, N_2 and H_2O uptakes in four PE-MOFs based on PACMOF charges and DDEC charges.

6. Topology distribution and its impact on pore features

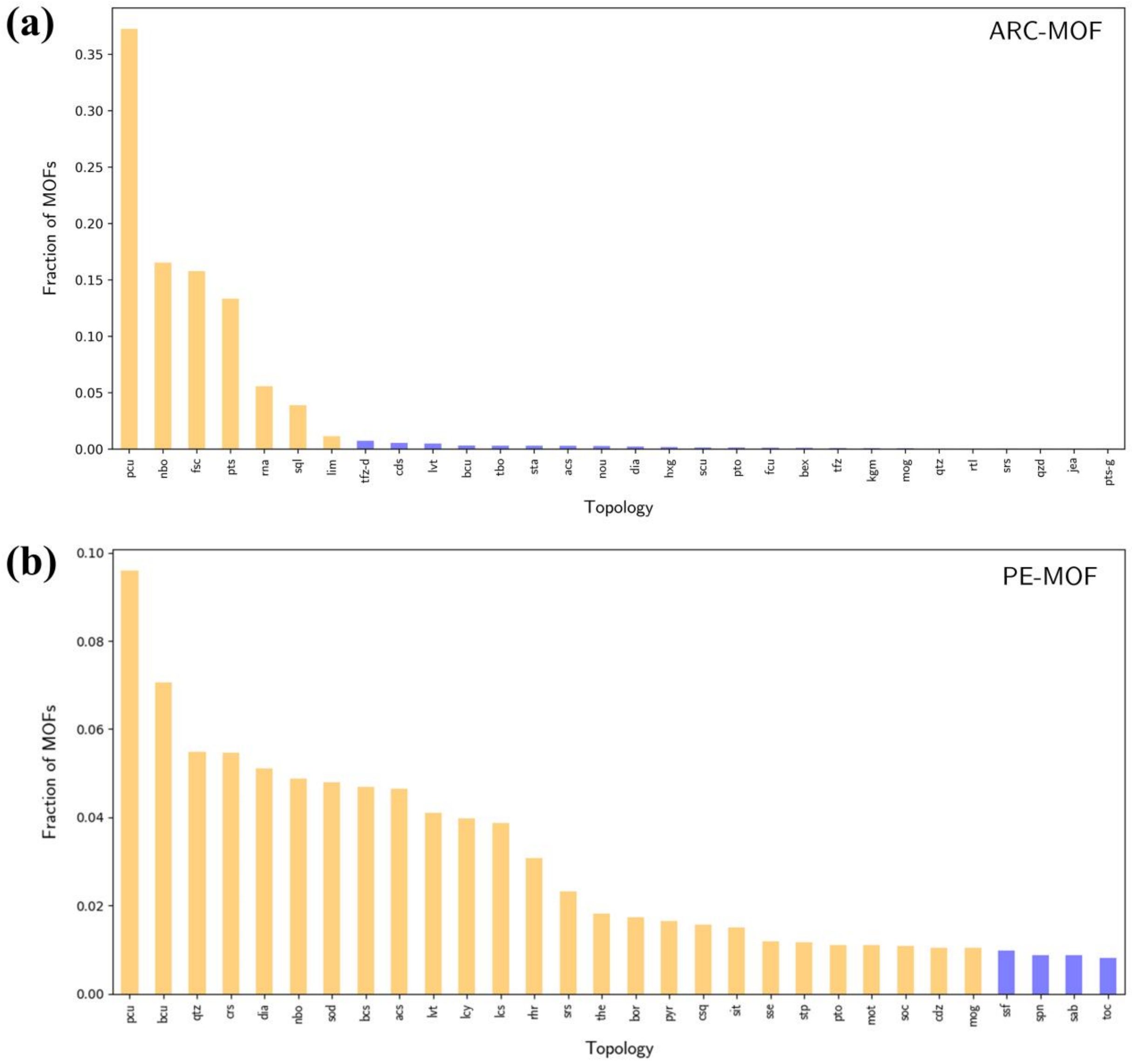

Fig. S8. Topology distribution in ARC-MOFs (top) and PE-MOFs (down). Topologies with more than 1% fraction are in orange, while those with less than 1% fraction are in blue.

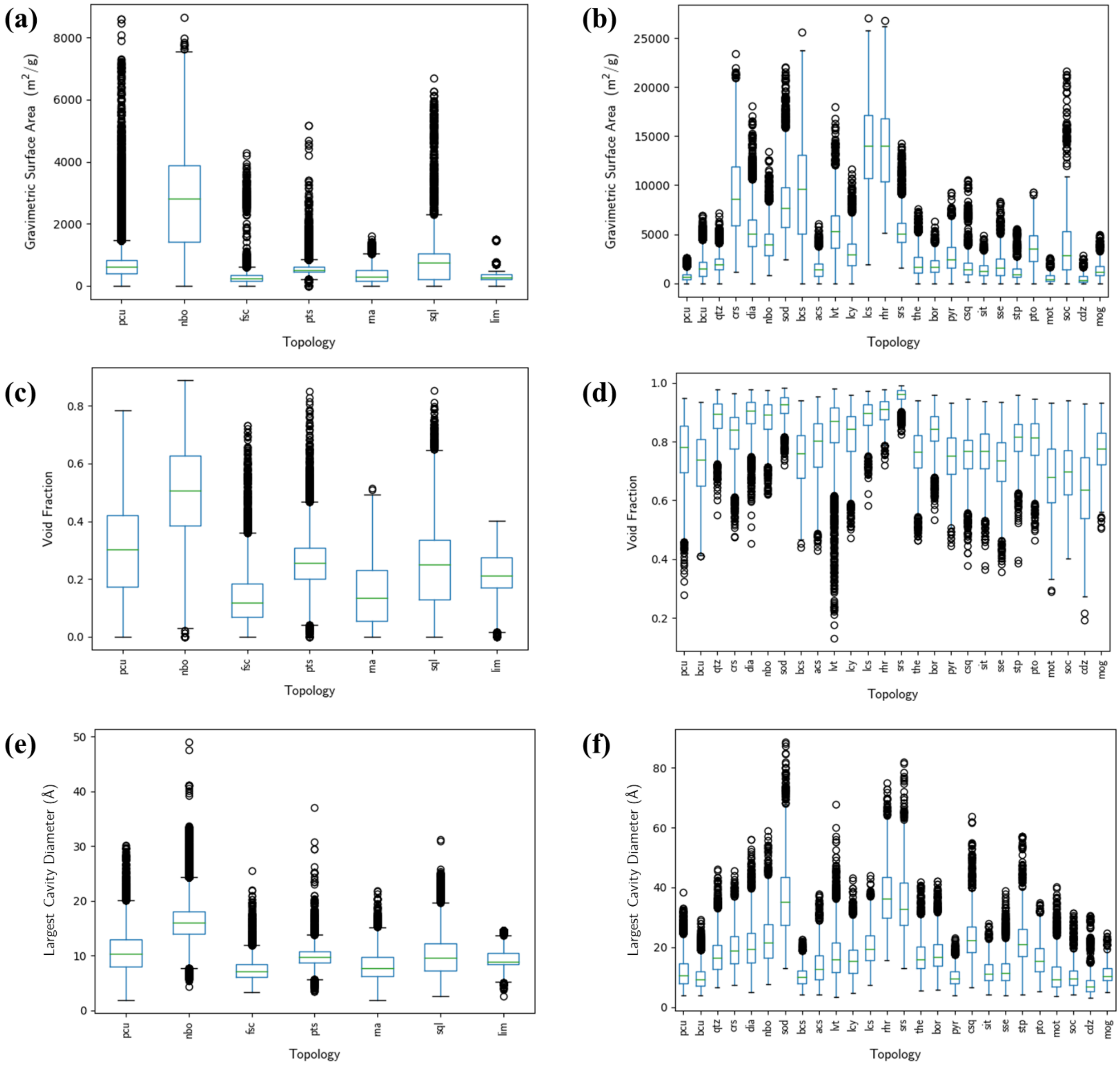

Fig. S9. Correlations of topology with pore features in ARC-MOFs (left) and PE-MOFs (right). Only topologies with more than 1% fraction are depicted.

7. t-SNE map of geometric features

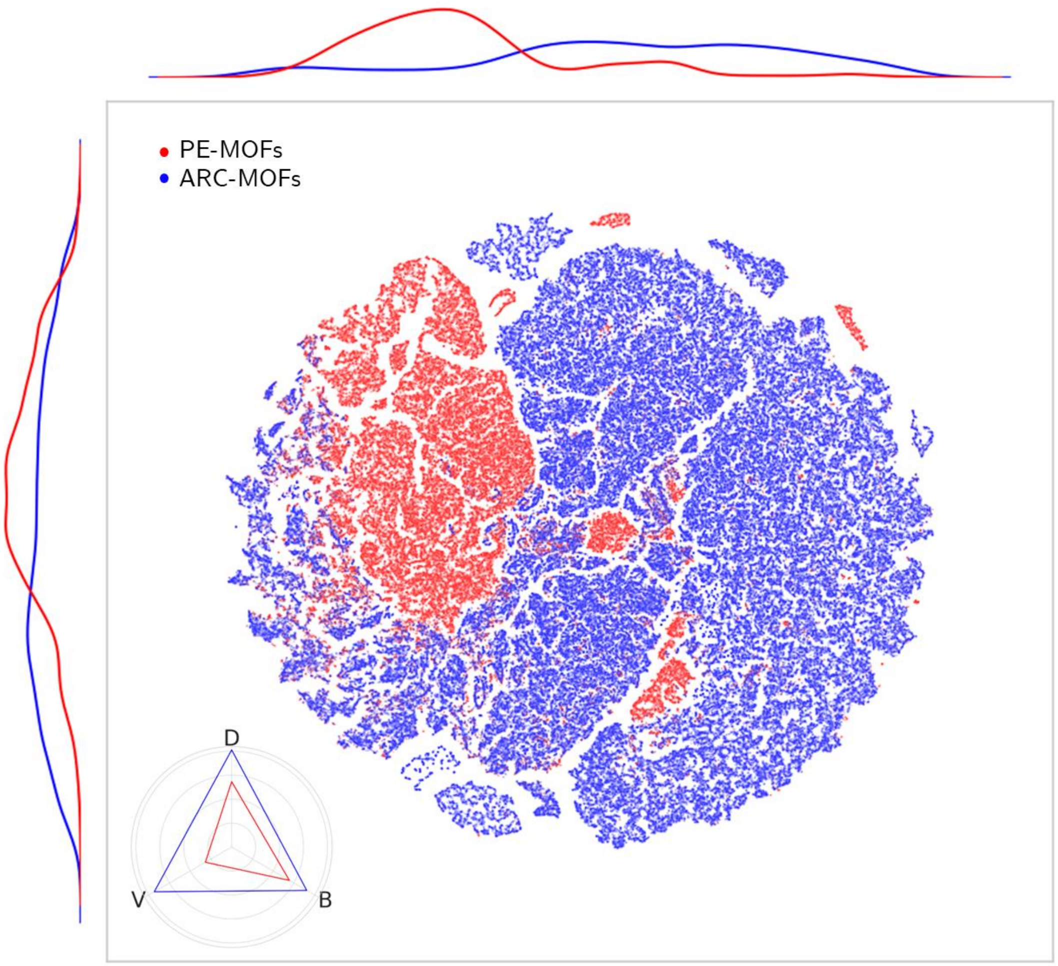

Fig. S10. t-SNE map of geometric features for 94,823 PE-MOFs (red) and 279,010 ARC-MOFs (blue). The curves along the axes are feature distributions. The radar chart displays diversity metrics of variety (V), balance (B) and disparity (D) in PE-MOFs (red) and ARC-MOFs (blue).

8. Comparative performance analysis of top-performing PE-MOF

Table S2. Performance of various materials for CO_2 capture.

Type	Material	N_{CO_2}	S_{CO_2/N_2}	Condition	Reference
Activated Carbon	PKC	4.67	28.99	1 bar, 298 K	8
Zeolite	CMS-A-5	3.21	35	1 bar, 298 K	9
ZIF	ZIF-DIA-3	6.18	-	1 bar, 298 K	10
PAF	PAF-1-CH_2NH_2	5.48	>1000	1 bar, 298 K	11
COF	TPE-COF-1	3.3	12.03	1 bar, 298 K	12
MOF	CALF-20	2.3	-	1 bar, 298 K	13, 14
	CALF-20 M-e	2	-		
	CALF-20 M-w	2.5	-		
MOF	ZnF(daTZ)	3.22	120	1 bar, 298 K	15
MOF	Mg/DOBDC	8.04	-	1 bar, 298 K	16
MOF	hMOF	7.66	275	1 bar, 298 K	17
MOF	hMOF	7.49	355	0.9 bar, 298 K	18
MOF	PE-MOF	8.28	346	1 bar, 298 K	This work

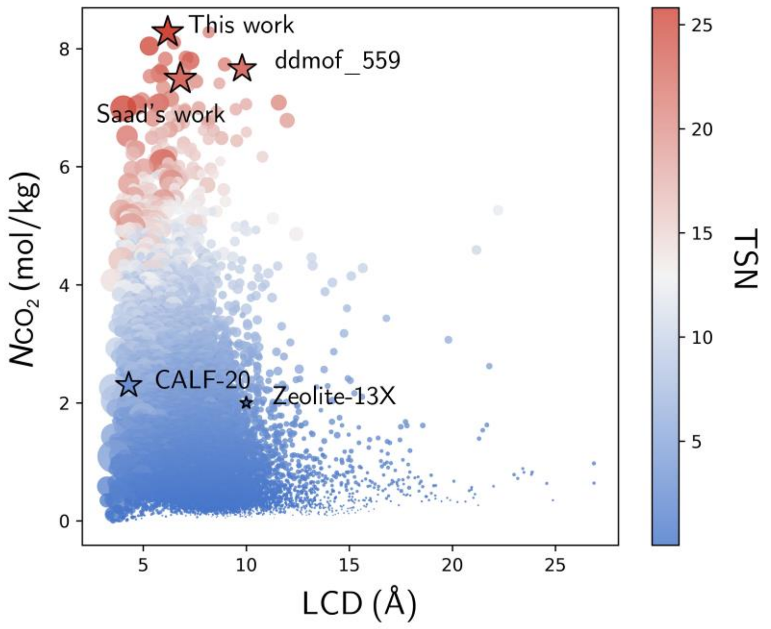

Fig. S11. Relationship between N_{CO_2} and LCD in top-performing MOFs from this work and previous studies.[13, 17-19]

9. Machine learning for CO_2 capture in PE-MOFs

Machine learning (ML) models were trained using the *Sci-kit learn* package.[20] A five-fold cross-validation was used to fine-tune the models with hyperparameters listed in **Table S3**. Bond information in primary BU have been successfully utilized in tree-based ML models for predicting the adsorption of small gas molecules such as CO_2 CH_4 and H_2.[21] In this work, along with basic pore descriptors, the type and number of bonds present in the BU were integrated, because textural features such as pore size and surface area were not embedded with chemical intuition. A full list of features is provided in **Table S4**.

Table S3. Hyperparameters for grid search.

Hyperparameters
n_estimators: [100, 200, 300, 500]
max_depth: [None, 10, 20, 30]
min_samples_split: [2, 5, 10]
min_samples_leaf: [1, 2, 4]

Table S4. Summary of features.

Set	Features
Pore	metal, GSA, VF, PV, POVF, POPV, Dimen, vertice, edge, topo, GCD, PLD, LCD
Bond (vertice)[a]	Ag-Ag (S) - m, Ag-C (S) - m, Ag-N (S) - m, Ag-O (S) - m, Ag-P (S) - m, Ag-S (S) - m, Al-Cl (S) - m, Al-N (S) - m, Al-O (S) - m, As-S (S) - m, B-B (S) - m, B-H (S) - m, B-N (S) - m, Ba-Cd (S) - m, Ba-O (S) - m, Be-O (S) - m, Bi-O (S) - m, Br-C (S) - m, Br-Cd (S) - m, Br-Cu (S) - m, Br-Zn (S) - m, C-C (A) - m, C-C (D) - m, C-C (S) - m, C-Cu (S) - m, C-F (S) - m, C-Ge (S) - m, C-H (S) - m, C-Ir (S) - m, C-N (A) - m, C-N (D) - m, C-N (S) - m, C-N (T) - m, C-O (A) - m, C-O (D) - m, C-O (S) - m, C-P (S) - m, C-Rh (S) - m, C-S (D) - m, C-S (S) - m, C-Sn (S) - m, C-Tb (S) - m, C-Zn (S) - m, Ca-Cd (S) - m, Ca-O (S) - m, Cd-Cd (S) - m, Cd-Cl (S) - m, Cd-F (S) - m, Cd-I (S) - m, Cd-K (S) - m, Cd-Mn (S) - m, Cd-N (S) - m, Cd-O (S) - m, Cd-S (S) - m, Ce-O (S) - m, Cl-Co (S) - m, Cl-Cu (S) - m, Cl-Fe (S) - m, Cl-In (S) - m, Cl-La (S) - m, Cl-Mn (S) - m, Cl-Nd (S) - m, Cl-O (S) - m, Cl-Pd (S) - m, Cl-Pt (S) - m, Cl-Rh (S) - m, Cl-Zn (S) - m, Co-Co (S) - m, Co-F (S) - m, Co-N (S) - m, Co-O (S) - m, Co-P (S) - m, Co-S (S) - m, Cr-N (S) - m, Cr-O (S) - m, Cs-In (S) - m, Cs-O (S) - m, Cu-Cu (S) - m, Cu-F (S) - m, Cu-I (S) - m, Cu-N (S) - m, Cu-O (S) - m, Cu-P (S) - m, Cu-S (S) - m, Cu-W (S) - m, Dy-N (S) - m, Dy-O (S) - m, Er-N (S) - m, Er-O (S) - m, Eu-Eu (S) - m, Eu-O (S) - m, F-Ge (S) - m, F-Nb (S) - m, Fe-Fe (S) - m, Fe-N (S) - m, Fe-O (S) - m, Fe-S (S) - m, Ga-N (S) - m, Ga-O (S) - m, Ga-S (S) - m, Gd-N (S) - m, Gd-O (S) - m, Ge-S (S) - m, H-N (S) - m, H-O (S) - m, H-S (S) - m, Hf-O (S) - m, Ho-N (S) - m, Ho-O (S) - m, I-Zn (S) - m, In-N (S) - m, In-O (S) - m, Ir-N (S) - m, K-O (S) - m, La-N (S) - m, La-O (S) - m, Li-O (S) - m, Lu-O (S) - m, Mg-O (S) - m, Mn-Mn (S) - m, Mn-N (S) - m, Mn-O (S) - m, Mn-S (S) - m, Mo-Mo (S) - m, Mo-N (S) - m, Mo-O (D) - m, Mo-O (S) - m, N-N (A) - m, N-

	N (D) - m, N-N (S) - m, N-Na (S) - m, N-Nd (S) - m, N-Ni (S) - m, N-O (D) - m, N-O (S) - m, N-Pb (S) - m, N-Pd (S) - m, N-Pt (S) - m, N-Rh (S) - m, N-Ru (S) - m, N-S (S) - m, N-Sn (S) - m, N-Sr (S) - m, N-Ti (S) - m, N-W (S) - m, N-Y (S) - m, N-Zn (S) - m, Na-O (S) - m, Nd-Nd (S) - m, Nd-O (S) - m, Ni-Ni (S) - m, Ni-O (S) - m, Ni-S (S) - m, O-O (S) - m, O-P (A) - m, O-P (D) - m, O-P (S) - m, O-Pb (S) - m, O-Pr (S) - m, O-Rh (S) - m, O-Ru (S) - m, O-S (A) - m, O-S (D) - m, O-S (S) - m, O-Sc (S) - m, O-Se (A) - m, O-Se (D) - m, O-Sm (S) - m, O-Sn (S) - m, O-Sr (S) - m, O-Tb (S) - m, O-Th (S) - m, O-Ti (S) - m, O-Tm (S) - m, O-U (D) - m, O-U (S) - m, O-V (S) - m, O-Y (S) - m, O-Yb (S) - m, O-Zn (S) - m, O-Zr (S) - m, P-Zn (S) - m, Pb-S (S) - m, Pr-Pr (S) - m, Rh-Rh (S) - m, Ru-Ru (S) - m, S-S (S) - m, S-W (S) - m, Th-Th (S) - m, Yb-Yb (S) - m, Zn-Zn (S) - m, B-X (S) - m, C-X (S) - m, N-X (S) - m, P-X (S) - m, S-X (S) - m
**Bond (edge)**	O-S (S) - o, C-X (S) - o, H-X (S) - o, O-X (S) - o, N-X (S) - o, B-C (S) - o, Br-C (S) - o, C-C (A) - o, C-C (D) - o, C-C (S) - o, C-C (T) - o, C-Cl (S) - o, C-F (S) - o, C-H (S) - o, C-I (S) - o, C-N (A) - o, C-N (D) - o, C-N (S) - o, C-N (T) - o, C-O (A) - o, C-O (D) - o, C-O (S) - o, C-P (S) - o, C-S (A) - o, C-S (D) - o, C-S (S) - o, C-Si (S) - o, F-S (S) - o, H-N (S) - o, H-O (S) - o, H-S (S) - o, N-N (A) - o, N-N (D) - o, N-N (S) - o, N-O (A) - o, O-P (D) - o, O-S (A) - o, O-S (D) - o

[a]X: dummy atom in BU linkage.

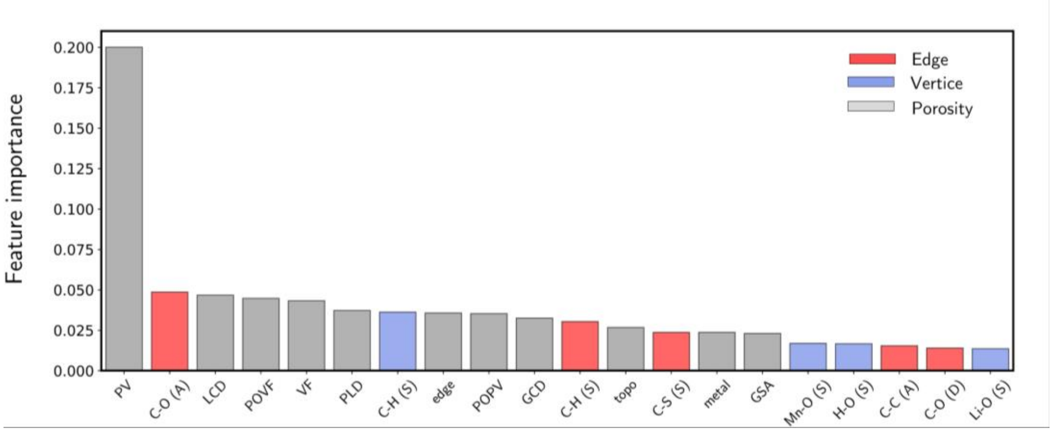

Fig. S12. Feature importance analysis for the 2nd ML model incorporating surrogate features.

References

(1) Padial, N. M.; Procopio, E. Q.; Montoro, C.; López, E.; Oltra, J. E.; Colombo, V.; Maspero, A.; Masciocchi, N.; Galli, S.; Senkovska, I.; et al. Highly Hydrophobic Isoreticular Porous Metal–Organic Frameworks for the Capture of Harmful Volatile Organic Compounds. *Angewandte Chemie* **2013**, *125*, 8448-8452.

(2) Gibaldi, M.; Kwon, O.; White, A.; Burner, J.; Woo, T. K. The HEALED SBU Library of Chemically Realistic Building Blocks for Construction of Hypothetical Metal–Organic Frameworks. *ACS Applied Materials & Interfaces* **2022**, *14*, 43372-43386.

(3) Burner, J.; Luo, J.; White, A.; Mirmiran, A.; Kwon, O.; Boyd, P. G.; Maley, S.; Gibaldi, M.; Simrod, S.; Ogden, V.; et al. ARC-MOF: A Diverse Database of Metal–Organic Frameworks with DFT-Derived Partial Atomic Charges and Descriptors for Machine Learning. *Chemistry of Materials* **2023**, *35*, 900-916.

(4) Gomez-Gualdron, D. A.; Gutov, O. V.; Krungleviciute, V.; Borah, B.; Mondloch, J. E.; Hupp, J. T.; Yildirim, T.; Farha, O. K.; Snurr, R. Q. Computational Design of Metal–Organic Frameworks Based on Stable Zirconium Building Units for Storage and Delivery of Methane. *Chemistry of Materials* **2014**, *26*, 5632-5639.

(5) Potoff, J. J.; Siepmann, J. I. Vapor–Liquid Equilibria of Mixtures Containing Alkanes, Carbon Dioxide and Nitrogen. *AlChE* **2001**, *47*, 1676-1682.

(6) Mayo, S. L.; Olafson, B. D.; Goddard, W. A. DREIDING: A Generic Force Field for Molecular Simulations. *Journal of Physical Chemistry* **1990**, *94*, 8897-8909.

(7) Rappé, A. K.; Casewit, C. J.; Colwell, K. S.; Goddard, W. A.; Skiff, W. M. UFF, A Full Periodic Table Force Field for Molecular Mechanics and Molecular Dynamics Simulations. *Journal of the American Chemical Society* **1992**, *114*, 10024-10035.

(8) Joshi, P.; Mehta, S.; Singh, N.; Dalakoti, S.; Divekar, S.; Dasgupta, S.; Srivastava, M.; Khatri, O. P. Fruit waste (pomelo peels)-derived activated carbons for biogas upgradation and capture of green house gases from flue gas and low concentration coalbed methane. *Journal of Environmental Chemical Engineering* **2023**, *11*, 110291.

(9) Wei, Y.; Zhao, T.; Wang, J.; Chen, Y.; Wang, Q.; Liu, X.; Zhao, Y. Ultramicroporous Carbon Molecular Sieve for Air Purification by Selective Adsorption Low-Concentration CO_2 and VOC Molecules. *Industrial & Engineering Chemistry Research* **2023**, *62*, 7635-7641.

(10) Lijuan, S.; Kai, Q.; Haonan, W.; Jie, L.; Mingyue, Q.; Qun, Y. In-situ amino-functionalization of zeolitic imidazolate frameworks for high-efficiency capture of low-concentration CO_2 from flue gas. *Fuel* **2021**, *298*, 120875.

(11) Zhang, P.; Zhang, C.; Wang, L.; Dong, J.; Gai, D.; Wang, W.; Nguyen, T. S.; Yavuz, C. T.; Zou, X.; Zhu, G. Basic Alkylamine Functionalized PAF-1 Hybrid Membrane with High Compatibility for Superior CO_2 Separation from Flue Gas. *Advanced Functional Materials* **2023**, *33*, 2210091.

(12) Altundal, O. F.; Altintas, C.; Keskin, S. Can COFs replace MOFs in flue gas separation? high-throughput computational screening of COFs for CO_2/N_2 separation. *Journal of Materials Chemistry A* **2020**, *8*, 14609-14623.

(13) Lin, J. B.; Nguyen, T. T. T.; Vaidhyanathan, R.; Burner, J.; Taylor, J. M.; Durekova, H.; Akhtar, F.; Mah, R. K.; Ghaffari-Nik, O.; Marx, S.; et al. A scalable metal–organic framework as a durable physisorbent for carbon dioxide capture. *Science* **2021**, *374*, 1464-1469.

(14) Wang, X.; Alzayer, M.; Shih, A. J.; Bose, S.; Xie, H.; Vornholt, S. M.; Malliakas, C. D.; Alhashem, H.; Joodaki, F.; Marzouk, S.; et al. Tailoring Hydrophobicity and Pore Environment in Physisorbents for Improved Carbon Dioxide Capture under High Humidity. *Journal of the American Chemical Society* **2024**, *146*, 3943-3954.

(15) Shi, Z.; Tao, Y.; Wu, J.; Zhang, C.; He, H.; Long, L.; Lee, Y.; Li, T.; Zhang, Y.-B. Robust Metal–Triazolate Frameworks for CO_2 Capture from Flue Gas. *Journal of the American Chemical Society* **2020**, *142*, 2750-2754.

(16) Caskey, S. R.; Wong-Foy, A. G.; Matzger, A. J. Dramatic Tuning of Carbon Dioxide Uptake via Metal Substitution in a Coordination Polymer with Cylindrical Pores. *Journal of the American Chemical Society* **2008**, *130*, 10870-10871.

(17) Majumdar, S.; Moosavi, S. M.; Jablonka, K. M.; Ongari, D.; Smit, B. Diversifying Databases of Metal–Organic Frameworks for High-Throughput Computational Screening. *ACS Applied Materials & Interfaces* **2021**, *13*, 61004-61014.

(18) Mohamed, S. A.; Zhao, D.; Jiang, J. Integrating stability metrics with high-throughput computational screening of metal–organic frameworks for CO_2 capture. *Communications Materials* **2023**, *4*, 1-10.

(19) Ho, M. T.; Allinson, G. W.; Wiley, D. E. Reducing the Cost of CO_2 Capture from Flue Gases Using Pressure Swing Adsorption. *Industrial & Engineering Chemistry Research* **2008**, *47*, 4883-4890.

(20) Pedregosa, F.; Varoquaux, G.; Gramfort, A.; Michel, V.; Thirion, B.; Grisel, O.; Blondel, M.; Prettenhofer, P.; Weiss, R.; Dubourg, V.; et al. Scikit-learn: Machine learning in Python. *Journal of Machine Learning Research* **2011**, *12*, 2825-2830.

(21) Bailey, T.; Jackson, A.; Berbece, R.-A.; Wu, K.; Hondow, N.; Martin, E. Gradient Boosted Machine Learning Model to Predict H_2, CH_4 and CO_2 Uptake in Metal–Organic Frameworks Using Experimental Data. *Journal of Chemical Information and Modeling* **2023**, *63*, 4545-4551.
